# Computationally guided bioengineering of the active site, substrate access pathway, and water channels of thermostable cytochrome P450, CYP175A1, for catalyzing the alkane hydroxylation reaction†

**DOI:** 10.1039/d3sc02857g

**Published:** 2023-12-04

**Authors:** Mohd Taher, Kshatresh Dutta Dubey, Shyamalava Mazumdar

**Affiliations:** a Department of Chemical Sciences, Tata Institute of Fundamental Research Homi Bhabha Road, Colaba Mumbai 400005 India chiraltaher@gmail.com shyamal@tifr.res.in; b Department of Chemistry, School of Natural Science, Shiv Nadar Institution of Eminence Delhi-NCR NH91, Tehsil Dadri Greater Noida Uttar Pradesh 201314 India kshatresh.dubey@snu.edu.in

## Abstract

Understanding structure–function relationships in proteins is pivotal in their development as industrial biocatalysts. In this regard, rational engineering of protein active site access pathways and various tunnels and channels plays a central role in designing competent enzymes with high stability and enhanced efficiency. Here, we report the rational evolution of a thermostable cytochrome P450, CYP175A1, to catalyze the C–H activation reaction of longer-chain alkanes. A strategy combining computational tools with experiments has shown that the substrate scope and enzymatic activity can be enhanced by rational engineering of certain important channels such as the substrate entry and water channels along with the active site of the enzyme. The evolved enzymes showed an improved catalytic rate for hexadecane hydroxylation with high regioselectivity. The Q67L/Y68F mutation showed binding of the substrate in the active site, water channel mutation L80F/V220T showed improved catalytic activity through the peroxide shunt pathway and substrate entry channel mutation W269F/I270A showed better substrate accessibility to the active pocket. All-atom MD simulations provided the rationale for the inactivity of the wild-type CYP175A1 for hexadecane hydroxylation and predicted the above hot-spot residues to enhance the activity. The reaction mechanism was studied by QM/MM calculations for enzyme–substrate complexes and reaction intermediates. Detailed thermal and thermodynamic stability of all the mutants were analyzed and the results showed that the evolved enzymes were thermally stable. The present strategy showed promising results, and insights gained from this work can be applied to the general enzymatic system to expand substrate scope and improve catalytic activity.

## Abstract 2

Rational design and engineering of the active pocket for access and binding of the substrate, and water channel in a thermostable cytochrome P450 enabled efficient catalysis of a longer chain alkane.

## Graphical abstract

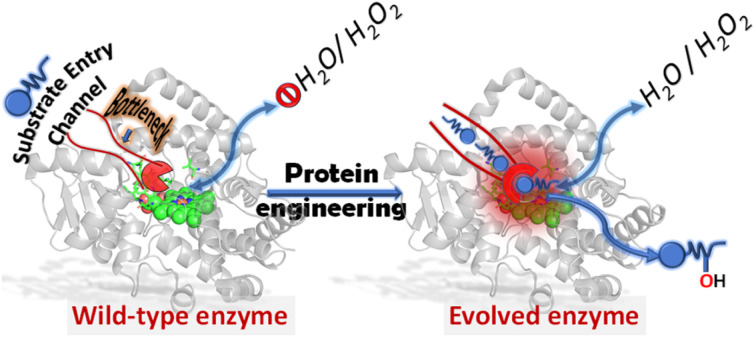

## 1 Introduction

Engineering and development of enzymes with new or improved catalytic activities are crucial for the advancement of biocatalysts for chemical and pharmaceutical industries.^1,2^ Protein engineering provides an eco-friendly and sustainable way to design new biocatalysts for promoting green chemistry. In recent years, advances in protein engineering strategies such as directed evolution by random mutagenesis and site-directed mutagenesis have paved the way to achieve this goal through the repurposing of natural enzymes. The directed evolution technique is the most commonly used approach, which involves screening of the active variants from a large library of mutants.^3^ Extensive studies are reported to design a ‘smart library’ of mutants to improve the efficiency of the directed evolution process.^4,5^ A structure-guided rational protein design approach could provide an efficient method to produce engineered enzymes with a minimal screening procedure and provides a better understanding of the structure–function relationship in the protein.^6^ Cytochrome P450s are amongst the most extensively studied enzymes and are considered as potential candidates for biotechnological applications.^7,8^ The engineered cytochrome P450 enzymes were shown to be more catalytically active and were also capable of catalyzing ‘new to nature’ chemical reactions such as C–C coupling^9^ and nitrogen insertion into an unactivated C–H bond.^10^ The rational evolution of the enzymes, has mostly been focused on active site engineering using site-directed and saturation mutagenesis,^11^ and also by using novel decoy molecules to expand the substrate scope.^12,13^ Modification of the metal cofactor^14,15^ along with active site mutation^16,17^ has also been investigated to catalyze abiological reactions by using this enzyme. In cytochrome P450s, the active site is deeply buried inside the core of the protein matrix and surrounded by a complex network of tunnels and channels which connect the active site with the protein surface. Tunnels in the cytochrome P450s serve as pathways for the substrate^18^ and other essential entities such as H_2_O, protons, and products to enter or exit the active site^19^ of the enzyme. These tunnels are lined with amino acid residues that are critical for substrate recognition and binding, as well as for controlling the flow of substrates and products through the enzyme.^20^ Hence, a study of the tunnels would provide better insight into the protein structure, and bioengineering of these tunnels may offer an efficient approach for the design of engineered enzymes with improved catalytic activity.^21^ The tunnels in cytochrome P450 enzymes are often highly specific and can discriminate between different substrates based on their size, shape, and chemical properties.^21,22^ The tunnels often contain aromatic amino acids, which act as gatekeepers for the incoming substrate.^23^ Improvement of the catalytic efficiency of cytochrome P450s and other enzymes by engineering some of these tunnels has been recently reported.^24–26^ However, the combined effects of rational design of the substrate binding site along with modification of important tunnels to expand the substrate scope and to increase the catalytic efficiency have not yet been reported. In the present work, we have used computational tools such as molecular docking and molecular dynamics simulation tools to complement the experiments for the development of engineered enzymes for a very challenging C^(sp^3^)^–H activation reaction. Due to its importance at the industrial scale, several researchers have attempted to develop enzymes for alkane hydroxylation.^27–30^ Arnold and co-workers developed cytochrome P450-based alkane hydroxylating enzymes with a remarkably high catalytic activity using the directed evolution technique.^31^ Using the decoy molecule strategy,^32^ Reetz and co-workers showed that CYP102A1 from *Bacillus megaterium*, also known as P450_BM3_ can be converted into an alkane hydroxylating enzyme.^33^ These findings were very promising, giving rise to high regio- and enantioselectivity, but still had several limitations such as low thermal stability of the enzyme, low tolerance towards organic solvents, and the use of the expensive electron donor NADPH. In a report, Cirino *et al.*^34^ engineered the P450_BM3_ enzyme to use cheaper and greener oxidants such as H_2_O_2_ for catalyzing the monooxygenation reaction as an alternate source of expensive NADPH, but the catalytic activity was very poor due to low coupling efficiency. To address these limitations, we have engineered the active site and two important tunnels *i.e.* the substrate entry channel and water channel in a thermostable cytochrome P450 from *Thermus thermophilus*, CYP175A1, to catalyze the alkane hydroxylation reaction through the peroxide shunt pathway using H_2_O_2_. CYP175A1 has a close sequence similarity with P450_BM3_. Like P450_BM3_, CYP175A1 has been shown to catalyze the fatty acid hydroxylation reaction^35^ indicating that both enzymes have similar active pocket sizes and environments. Hence, we anticipate that CYP175A1 could be a thermostable analog for P450_BM3_. Wild-type CYP175A1 does not show any detectable catalytic activity for the alkane hydroxylation reaction. For proof of the concept, we selected hexadecane as the alkane substrate to investigate the alkane hydroxylation activity in the variants of the enzyme. Hexadecane has a close structural resemblance with palmitoleic acid (hexadecane-9-enoic acid), which was previously shown to efficiently bind inside the active pocket of the wild-type enzyme with the long hydrophobic tail residing inside the active site.^35^ We employed a simple molecular docking tool to understand the probable substrate orientations inside the active pocket of the enzyme and selected the lowest energy binding pose of the enzyme–substrate complex to identify the important residues (hotspots) in the substrate binding site for mutagenesis. This helped us create a virtual library of mutants, which was screened using molecular docking to identify the most suitable mutant. We have selected the most suitable mutant from the analysis of the docking results, and performed all-atom MD simulations on the substrate-bound structure of the mutant. The MD simulation data analysis helped us to further assess the stability of the enzyme–substrate complex in the presence of segmental dynamics. The MD simulations further suggested that there is a bottleneck at the substrate entry channel, and the water channel is also not properly formed at the active site of this mutant, which could result in lower activity. Tunnel analyses using CAVER^36^ have also suggested the presence of a bottleneck at the substrate entry channel. Guided by the MD simulation data we made mutations to activate the ‘water channel’ inside the protein matrix and also modified the residues at the bottleneck of substrate entry. The design of the mutant enzymes based on the MD simulations and docking analysis was validated experimentally. The experimental results show good agreement with the computational results. Furthermore, QM/MM calculations were carried out on the mutant enzyme to understand the reaction mechanism and energetics. The results showed that the developed mutant follows the typical cytochrome P450 reaction mechanism for C–H activation.

## 2 Results and discussion

**Scheme 1:**
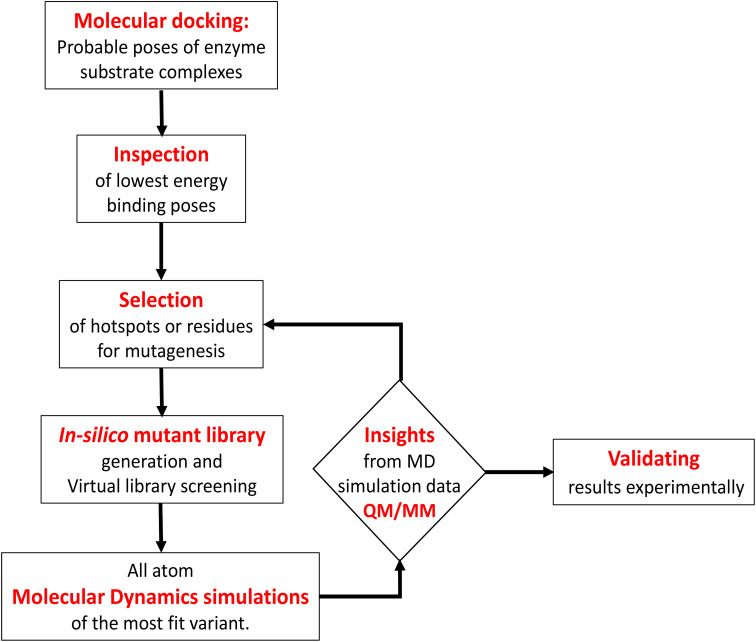
Flowchart of computationally guided design of an alkane hydroxylating enzyme.

**Fig. 1:**
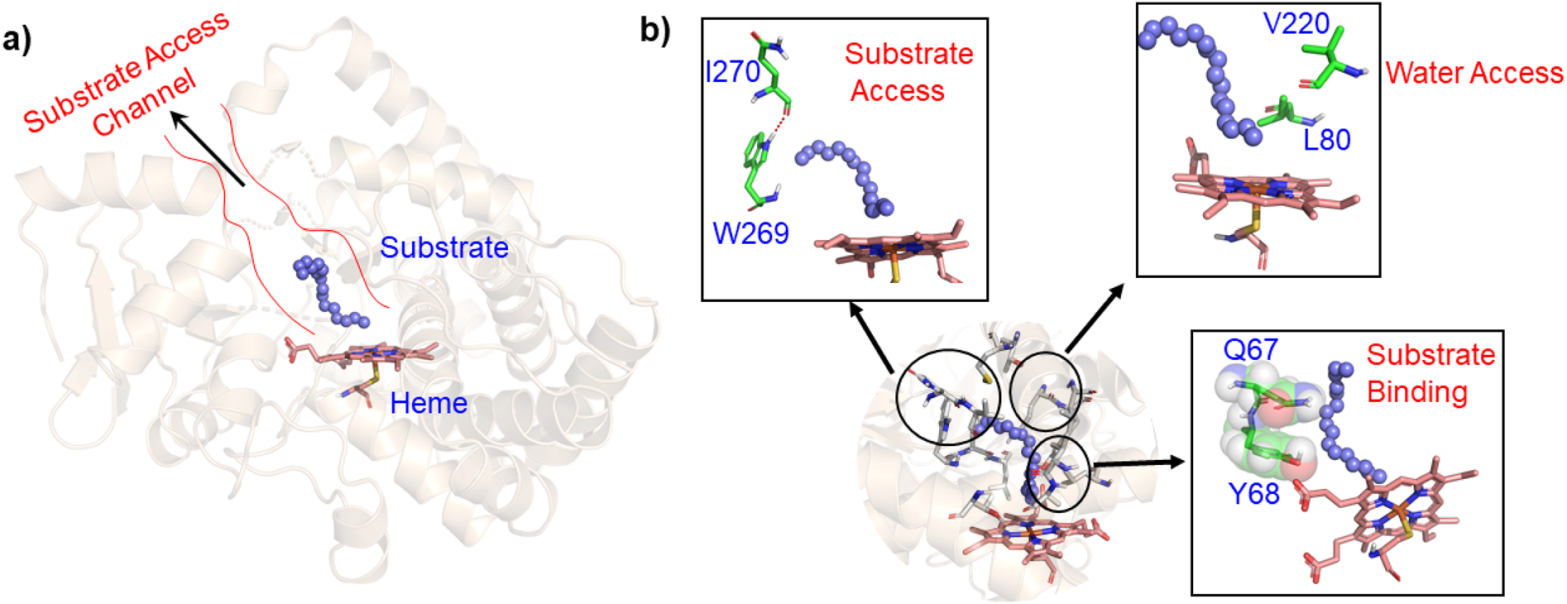
(a) Lowest energy docking pose of wild-type CYP175A1 with hexadecane (blue balls), and the substrate access channel is highlighted by red lines. (b) Strategic residues which may affect catalytic activity. Note the blockage of the substrate access channel by H-bonds between I270 and W269, and steric blockage by Q67-Y68 for substrate binding.

In contrast to P450_BM3_, which is widely used as a scaffold for various bioengineering studies,^37–39^ the active site architecture of CYP175A1 is less elucidated. We, therefore, started our study to understand the active site topology of CYP175A1 vis-à-vis CYP102A1 (*i.e.*, P450_BM3_). In doing so, we first superimposed the crystal structures of CYP175A1 (PDB ID: 1N97)^40^ with those of CYP102A1 (PDB ID: 1FAG).^41^ Superimposition shows that both structures have almost identical tertiary structural folds with an RMSD of 1.07 Å (Fig. S1 in the ESI†). Cavity analysis using Pymol showed that the active site of CYP175A1 is reasonably large and is of U shape (Fig. S2 in the ESI†) geometry. Similar to cytochrome P450_BM3_, the active site of CYP175A1 contains mostly hydrophobic amino acids (Table S1 in the ESI†). These data suggested that longer-chain alkanes can easily be accommodated inside the active pocket of CYP175A1. However, screening of medium to long-chain alkanes for hydroxylation by wild-type CYP175A1 shows no detectable activity, and therefore, we aim to bioengineer the CYP175A1 enzyme for C–H hydroxylation using computational guided rational designing. We have taken hexadecane as a substrate to start rational design using a strategy highlighted in Scheme 1. Initially, hexadecane was docked into the active pocket of wild-type CYP175A1 (see Fig. S3 and S4 and section 6 in the ESI† for details), and the best pose based on the lowest energy score was chosen. Fig. 1a shows the lowest energy pose of hexadecane docked into the active pocket of the wild-type (WT) enzyme, while Fig. 1b shows some strategic residues close to the active site. Based on the docked structure of the substrate (Fig. 1b) we adopted three key strategies during bioengineering:

### 2.1 Substrate binding engineering

To understand the rationale behind the absence of any detectable catalytic activity of the WT enzyme, we performed the MD simulation on the lowest energy-docked structure of the substrate–enzyme (WT) complex. To our surprise, we found that the substrate was ejected out of the binding pocket of the enzyme during the course of the simulation (see supplementary video number VS1†) signifying that the active pocket of the WT enzyme is not suitable for substrate binding. A closer inspection of the MD trajectory reveals that active site residues Q67 and Y68 provide steric repulsion on the substrate that leads to unbinding of the substrate. The presence of Q67 residue hindering substrate (palmitoleic acid) binding has also been proposed earlier by Poulos and co-workers.^40^ In addition, the polar group of these residues interacts with the propionate of heme, which provides stiffness to the active site, and therefore, we anticipate that the replacement of these polar residues with appropriate non-polar residues could increase the binding of hexadecane. With these insights, we prepared a library of double mutants of Q67 and Y68 by replacing these two residues with non-polar residues and docked the substrate in the active site. Interestingly, the docking/virtual screening data (Tables S2, S3, and Fig. S5 in the ESI†) of the substrate into the double-mutant variants showed an enhanced docking score relative to that of the WT complex, which indicated that replacement of Q → L and Y → F (*i.e.*, Q67L/Y68F termed an LF mutant) could be promising for hexadecane binding. To further substantiate the docking result, we performed the MD simulation of the best-docked pose of the substrate in complex with the LF variant of CYP175A1. Surprisingly, in contrast with the WT simulation, where the substrate came out of the binding pocket; the substrate shows stable binding with the LF mutant, and it was not ejected out of the binding pocket (supplementary video VS2†) during MD simulation. It is important to note here that the formed hydroxylated product will be polar and hence will have lower binding affinity compared to the substrate due to which another incoming substrate molecule can easily replace the product from the active pocket during turnover.

**Scheme 2:**
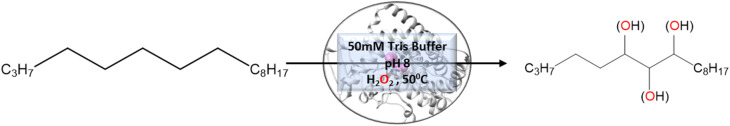
Hexadecane reaction catalysed by mutants of CYP175A1 through the peroxide shunt pathway.

To validate the above computational prediction, we performed LF mutation using site-directed mutagenesis by PCR and measured the experimental catalytic efficiency. The mutant protein was expressed and purified according to the previously reported protocols^42^ with minor modifications (see the ESI† for details). The purified protein showed a characteristic peak at 450 nm in a reduced CO-bound state (Fig. S9 in the ESI†). Purified mutant proteins were used to catalyze the hexadecane hydroxylation reaction as shown in Scheme 2. The mutant protein showed moderate catalytic activity (TON = 52) towards hexadecane through the reductive pathway but only a trace level of activity through the peroxide shunt pathway using H_2_O_2_ (Tables 1 and 2 in the ESI† for more details). It is important to note here that the wild-type enzyme was not showing any detectable catalytic activity for hexadecane. Our designed double mutant LF showed catalytic activity for hexadecane hydroxylation, which reflects that the substrate is indeed binding inside the active pocket of the LF mutant and also that the environment inside the active pocket of the wild-type enzyme was not favorable for highly non-polar substrates such as hexadecane. In the LF mutant we have incorporated small hydrophobic amino acids, which possibly increase the hydrophobicity and the accessibility of the active pocket, and hence, a bulky substrate like hexadecane showed binding inside the active pocket. Lower activity through the shunt pathway might be either due to irreversible inactivation of the protein by H_2_O_2_ or poor coupling of H_2_O_2_ with the protein active pocket, as has also been observed in other reports.^43^

**Table 1:** Details of sequential mutations on CYP175A1 by the multi generation approach

Generation	Mutant description	Mutation sites and corresponding mutations	Abbreviation
1st	Active site mutation	Q67L/Y68F	LF
2nd	Water channel activation mutations	L80F/V220T	FT
3rd	Substrate entry channel opening mutations	W269F/I270A	FA
4th	Water channel + substrate entry	L80F/V220T/W269F/I270A	FTFA
5th	Active site + substrate entry	Q67L/Y68F/W269F/I270A	LFFA
6th	Active site + water channel	Q67L/Y68F/L80F/V220T	LFFT
7th	Active site + substrate entry + water channel	Q67L/Y68F/V220T/W269F/I270A	LFTFA
8th	Combining all	Q67L/Y68F/L80F/V220T/W269F/I270A	LFFTFA

**Table 2:** Evaluation of catalytic activity: product distribution by various evolved enzymes for hexadecane hydroxylation^a^

Evolved enzyme	TON w.r.t the FA (W269F/I270A) mutant	Product distribution^b^			
		6-OH	7-OH	8-OH	Others^c^
Wild-type	N.D.	—	—	—	—
LF	2	8	92	—	—
FA	1	13	83	4	—
FT	N.D.	—	—	—	—
FTFA	5	61	37	2	—
LFFT	8	10	82	8	—
LFFA	12	17	80	2	<1
LFTFA	15	2	92	4	<3
LFFTFA	19	2	95	1	<2

a Catalytic activities are reported through the peroxide shunt pathway. The reaction was performed for 24 hours.

b Product distribution (in percentage) was quantified by GC-FID at least in triplicate. The error was less than 5%.

c Unidentified product peaks. N.D. stands for not determined.

### 2.2 Water channel engineering

**Fig. 2:**
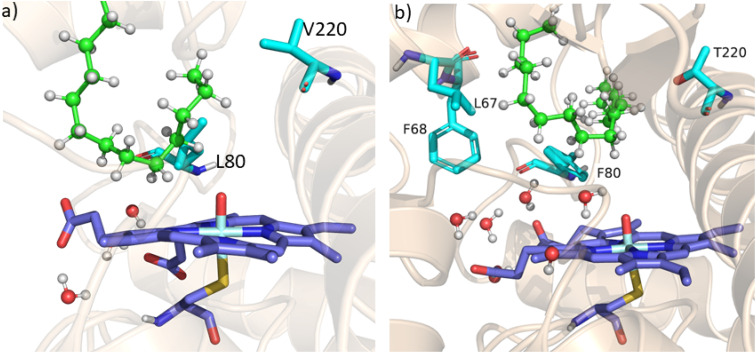
(a) Figure showing low water content inside the pocket of the LF mutant of CYP175A1. (b) Figure showing high water content inside the active pocket of the LFFT mutant of CYP175A1 (see supporting video VS3 for more details;† images taken from MD simulation data).

As mentioned above, the double mutant LF has enhanced the catalytic activity compared to the WT enzyme. However, its catalytic activity was ‘moderate’ and insufficient to be a practical, robust catalyst through the peroxide shunt mechanism. A recent study by Shaik and co-workers^44^ highlighted the importance of water molecules close to the heme site in the C–H activation reaction. We anticipated that as water and H_2_O_2_ are very much similar and completely miscible, activating the water channel may enhance the abundance of the H_2_O_2_ near the heme active site, and thus may increase the reaction rate of the peroxide-mediated C–H activation pathway. We, therefore, focused on the water channel inside the active pocket of CYP175A1 and hypothesized that enhancing hydration close to the heme site may increase the catalytic activity through the peroxide shunt pathway. The analysis of MD trajectories of the WT and LF mutant shows very low water occupancy throughout the simulations as shown in Fig. 2a (see Fig. S7 and supporting video VS2†). Through a closer inspection of the protein topology and MD trajectory of the WT enzyme, we found that L80 and V220 are two residues that may block the water access to the heme site. Therefore, we mutated V220 by using a threonine (Thr) residue since Thr is known for its role in oxygen activation and water channel formation in cytochrome P450 chemistry.^45–47^ Earlier studies on cytochrome P450_BM3_ showed that a phenylalanine residue close to the heme site plays the role of gatekeeper and can regulate the water aqueduct and substrate orientations.^48,49^ Inspired by these findings, we mutated L80 → F80 by assuming that Phe can play a gatekeeper role in substrate/water access since L80 in CYP175A1 occupies an equivalent position to that of Phe87 in cytochrome P450_BM3_. Interestingly, the MD simulation of this new tetra mutant incorporating mutations in two key areas *i.e.*, the substrate binding site and water channel site (Q67L/Y68F/L80F/V220T, termed the LFFT mutant) showed a significant enhancement of the water occupancy in the first hydration shell of the heme center as shown in Fig. 2b. The computationally predicted mutation of L80F/V220T was further verified by an experimental study, and we found that L80F and V220T mutations on the LF mutant (resulting in the LFFT mutant) were able to increase the catalytic activity through the peroxide shunt pathway, as shown in Fig. 4. A recent study by Zhao *et al.* showed that the peroxygenase activity of CYP199A4 and CYP153_AM.aq_ can be increased by rational engineering of the water channel,^50^ and closely agrees with our results.

### 2.3 Substrate access engineering

**Fig. 3:**
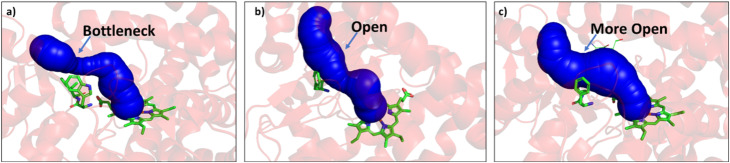
(a) Figure showing the bottleneck at the substrate entry channel in the wild type CYP175A1 using CAVER. (b) W269F/I270A double mutant of wild type CYP175A1 shows appreciable opening of the bottleneck at the substrate entry channel. (c) Q67L/Y68F/W269F/I270A tetra mutant of wild type CYP175A1 showing the opening of the bottleneck at the substrate entry channel.

Since easy access of the substrate to the active pocket and efficient product exit are crucial for catalysis, theoretical as well as experimental studies on understanding the substrate access channels in various cytochrome P450s have been performed previously, and it has been established that the catalytic activity can be influenced by how the substrate approaches the active site through the entry channel.^26,51^ We envisioned that catalytic activity could be improved by increasing the substrate accessibility to the active pocket. We therefore, focused on the engineering of the substrate access channel. We used the CAVER software tool to analyze the tunnel inside the crystal structure of the WT CYP175A1. CAVER tunnel analysis showed that the substrate entry tunnel has a bottleneck mainly in the vicinity of the W269 and I270 residues, as shown in Fig. 3. We substituted these residues with smaller residues of a similar type and analyzed the tunnel size using CAVER followed by docking of hexadecane into the mutant enzymes. Based on the good docking score and wider neck of the channel, we identified W269F and I270A as the best mutants as shown in Fig. 3. The binding of the substrate in the W269F/I270A mutant was further verified by MD simulations, and we found the substrate was quite stable inside the active site during the MD simulation as well.

**Fig. 4:**
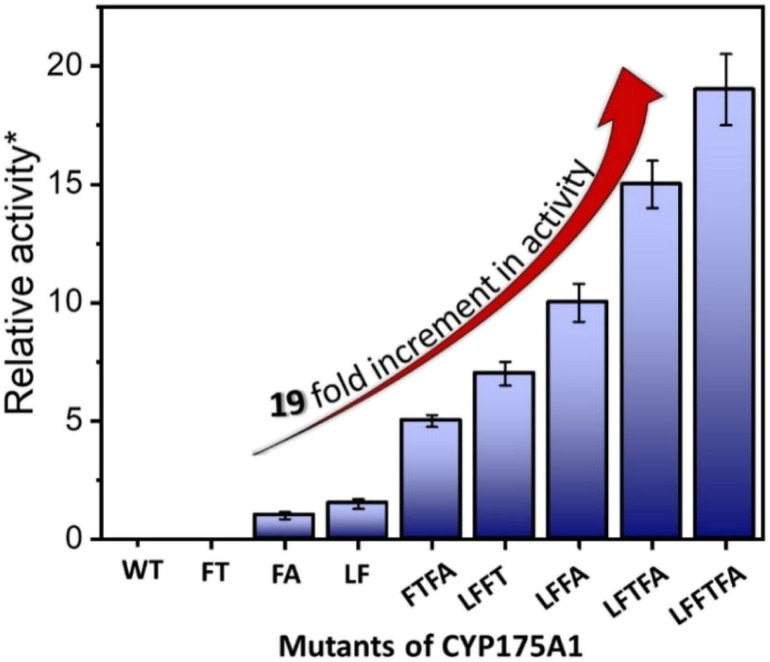
Hexadecane hydroxylation activity of the mutants of CYP175A1 through the peroxide shunt pathway using H_2_O_2_. *Relative fold increment in the TON with respect to the least performing FA mutant.

In brief, the simulations identified three strategic sites/hot spots for mutations in the WT enzyme based on: (1) substrate binding mutation, (2) water channel activation, and (3) substrate entry channel. Table 1 summarises these functional sites and the mutants we have made in this study. Mutations were performed using PCR (Table S6†) and validated by plasmid sequencing results (Fig. S8 in the ESI†). All the mutant proteins showed a characteristic CO binding spectrum under reduced conditions (Fig. S9 in the ESI†). To validate the computation prediction, we experimentally verified the catalytic efficiency of these mutants through the peroxide shunt pathway (see Fig. 4, S10–15, and section 21 in the ESI†). As can be seen in Fig. 4, the water channel mutant (FT) alone didn't show any detectable catalytic activity. This signifies that the substrate probably does not bind inside the active pocket due to an unfavorable environment for the hexadecane substrate. The LF mutant showed better activity compared to the FA mutant which signifies that substrate binding mutations (Q67L/Y68F) are more effective and crucial compared to substrate entry channel mutations (W269F/I270A). Due to the opening of the bottleneck, the FA mutant alone showed a small enhancement of activity. As shown in Fig. 4, hexadecane hydroxylation activity gradually started increasing upon sequential mutations signifying the synergistic effect of all the mutations necessary for the enhanced catalytic activity. This signifies that rational bioengineering is an effective way to expand the substrate scope of the enzyme. Furthermore, we were successfully able to achieve a 19-fold increase in the catalytic activity in the final mutant LFFTFA compared to the least-performing mutant FA. This signifies that rational bioengineering can not only expand the substrate scope of an enzyme but can also improve the catalytic activity. For a comparison, our developed final mutant performs better than the other hexadecane hydroxylating cytochrome P450 CYP52A4 reported earlier.^52^ These findings signify that engineering of all the hot spots in an enzyme is necessary for higher catalytic activity. The cumulative effect of all the mutations has significantly increased the catalytic reaction rate. The effect of mutations was also significant on the product distribution as evident from Table 2. The LF mutant showed 92% formation of the C7 hydroxylated product and the FA mutant showed 83% formation of the C7 hydroxylated product. The FTFA mutant has C6 hydroxylation as the major product (61%), which is probably due to the displacement of the substrate towards the entry channel by L80F/V220T mutation. There were some other unidentified peaks on the GC chromatograph possibly corresponding to the overoxidation product (see Fig. S15 in the ESI†) formed during the reaction.

### 2.4 Computational analysis by QM/MM studies on the mutants of CYP175A1

**Fig. 5:**
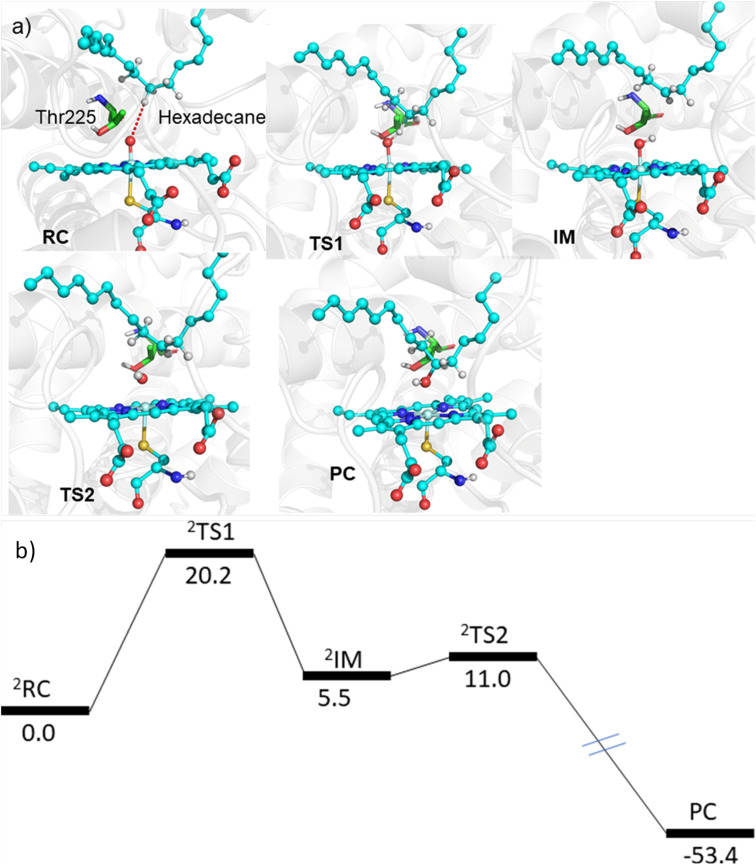
(a) The geometry of different stationary points during the hydroxylation of hexadecane *via* compound I inside the active pocket of the LFFTFA mutant, (water molecules near the active site of the protein are not shown for the ease of representation). (b) The energy profile of the reaction obtained from QM/MM potential energy surface scanning using the UB3LYP-D3/def2-TZVP level of the theory. All energy values are in kcal mol^−1^ and the energy for PC is not scaled.

The MD simulations combined with the experiments show that the mutants can efficiently catalyze the C–H hydroxylation of hexadecane. However, classical simulations cannot explain the reaction mechanism, so we performed hybrid QM/MM calculations to validate the reaction mechanism of C–H hydroxylation. For doing so, we selected a representative snapshot from the MD simulation of the most efficient variant, *i.e.*, LFFTFA, and performed the QM/MM optimization of the reactant geometry. Compound I (RC) mediated P450 reactions can be observed in doublet and quartet spin states. Earlier studies have shown that the doublet spin state is the favoured one.^47,53^ The calculations were performed using the B3LYP/def2-TZVP level of theory (See the ESI† the QM/MM calculation protocol). The calculated geometry of RC, TS1, IM, TS2 and PC corresponding to different stationary points during the hydroxylation of hexadecane *via* compound I (RC) inside the active pocket of the LFFTFA mutant of CYP175A1, is shown in Fig. 5. As can be seen in Fig. 5 (and Fig. S6, Tables S4 and S5 in the ESI†), the process starts from the hydrogen atom transfer (HAT) reaction from the C7 position of the substrate to form an unstable intermediate IM. The spin density calculations confirm the species IM as Cpd II as mentioned in earlier studies.^47,53^ The energy barrier of this reaction is 20.2 kcal mol^−1^, which is higher than the energy barrier for HAT for the reaction of fatty acid catalyzed by other cytochrome P450s^44,54^ reported earlier. In the subsequent steps, IM follows the typical rebound mechanism^55^ to form the hydroxylated product (PC) *via* a relatively small energy barrier of 5.5 kcal mol^−1^ and resets the catalytic cycle at the resting state for the next turnover of catalysis. The low energy barriers observed in the computational studies for the reactions possibly signify that the diffusion of the substrate to the active site is the rate-limiting step for the C–H activation reaction. The reaction proceeds to completion very rapidly as soon as the substrate reaches the active site. The theoretical calculations on the analogous fatty acid substrates were reported previously and they agree well with the present results.^56^ Furthermore, we also checked the reaction profile in the quartet spin state, and the reaction profile for the same is shown in Fig. S6 (in the ESI†). As can be seen in Fig. S6 (in the ESI†), the energy barrier for HAT from the RC in the quartet spin state is significantly higher than that of the doublet state (23.9 kcal mol^−1^ in the quartet *vs.* 20.2 kcal mol^−1^ in the doublet); therefore, we believe the reaction will proceed *via* the doublet state as reported earlier.^47,53^

### 2.5 Detailed characterization and evaluation of the thermal stability of the mutants of CYP175A1

**Fig. 6:**
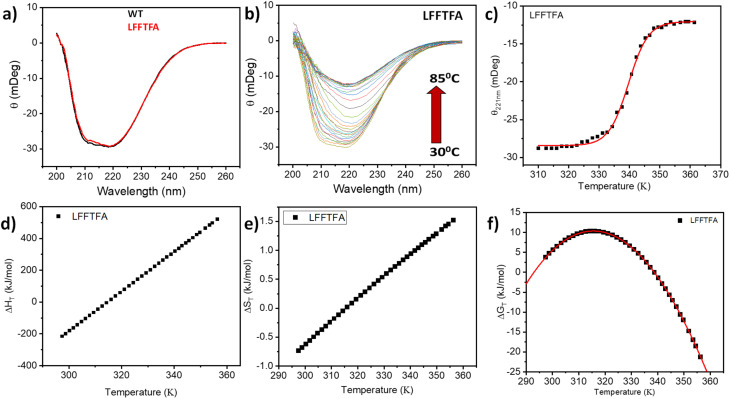
(a) CD spectrum of wild-type (WT) CYP175A1 (black) and the evolved variant LFFTFA of CYP175A1 (red) in the far UV region. (b) Thermal unfolding of the LFFTFA mutant: CD spectrum at different temperatures from 30 °C to 85 °C. (c) Plot of the CD signal (at 220 nm) at different temperatures of the LFFTFA mutant. (d) Enthalpy change (e) entropy change and (f) free energy change during thermal denaturation of the LFFTFA mutant.

**Fig. 7:**
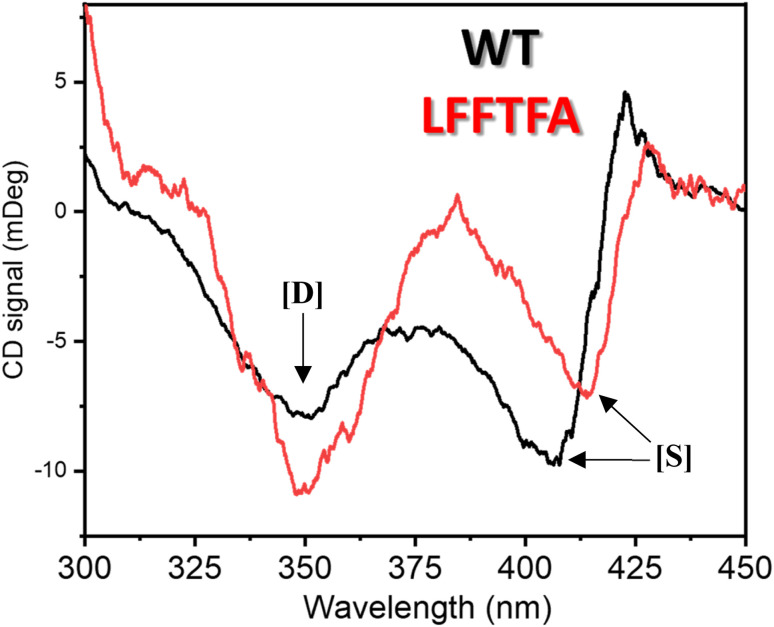
CD spectrum of substrate free wild-type (black) and the LFFTFA mutant (red) of CYP175A1 in the 300–450 nm region showing the Soret and delta bands. [*D*] denotes the delta band and [*S*] denotes the Soret band.

Reports on enzyme design by directed evolution through random mutagenesis have produced highly catalytically active evolved enzymes, but most often these reports lack detailed characterization of the evolved enzyme. To have a practical biocatalyst for industrial application, the evolved catalyst should be stable. We evaluated the detailed thermal stability of the evolved variants of CYP175A1 using circular dichroism (CD) spectroscopy. The CD spectrum for the secondary structure region (200–260 nm) of the wild type and mutants of CYP175A1 was recorded at 50 °C. The results showed that all the mutants were well folded and had a similar secondary structure to that of the wild-type protein. Fig. 6a shows the CD spectrum of the wild-type and LFFTFA mutant of CYP175A1, and overlapping spectra signify that there is not much appreciable change in the secondary structure of the protein upon mutations. The thermal unfolding of all the mutant proteins was also studied by recording the CD spectrum at different temperatures. All the temperature-dependent CD spectra were recorded from 30–85 °C, and Fig. 6b shows the thermal unfolding of the LFFTFA mutant. As shown in Fig. 6c, for the LFFTFA mutant, the CD signal at 221 nm was plotted as a function of temperature, and the data were analyzed using modified Gibbs Helmholtz equations.^57^ The values of the thermodynamic parameters obtained from the analyses of the data for all the mutants are given in Table S7 in the ESI.† Analysis of the data showed that the midpoint temperature, Tm for the unfolding of the secondary structure of the mutants of CYP175A1 has decreased compared to that of the wild-type protein but still the final mutant *i.e.* LFFTFA having a Tm value of 66 °C was found to be more thermally stable than the mesophilic analogs such as CYP101A1 and CYP102A1. Every mutation in the wild-type protein has led to a decrease in the overall thermal stability of the protein (Fig. S16 and Table S7 in the ESI†). To probe the effect of individual amino acids on the thermal stability of the protein, we have analyzed the thermal unfolding data of all the mutants. The decrease in the thermal stability of the QY mutant was attributed to the loss of hydrogen bonding due to the mutation of Y68 with Phe because the Y68 residue exhibited hydrogen bonding with the heme group and surrounding amino acids as understood from the crystal structure of the wild-type CYP175A1 (Fig. S17 in the ESI†). Interestingly the temperature of maximum stability (the temperature at which the slope of the free energy curve is zero, Fig. 6f) for the LFFTFA mutant is increased to around 33 °C compared to that of wild-type protein which has a temperature of maximum stability of 29 °C. This suggests that the final mutant is still thermally very stable and can work very well under ambient temperature conditions. The CD spectrum in the visible region (Fig. 7) was analyzed to probe the effect of the mutations on the tertiary structure of the protein. The results showed a distinct shift in the position of the Soret band (413 nm) in the LFFTFA mutant compared to that o the wild-type protein (408 nm), whereas there was no shift in the delta band position.

The Soret to delta band ratio (*S*/*D*) in the LFFTFA mutant was found to be (0.66) lower than that of the wild-type protein signifying the reduction in the polarity and hydrogen bonding near the active site of the protein. These observations have also been reported previously and agree well with the previous literature.^57–59^Table 3 shows the position and the ratio of the Soret and delta bands in various cytochrome P450s. Reduction in the *S*/*D* in LFFTFA as shown in Table 3 signifies that the active pocket is highly non-polar and perfect for the incorporation of non-polar substrates such as linear chain alkanes.

**Table 3:** Active site spectral features of various cytochrome P450s in the substrate-free state, showing the position of Soret and delta bands and their ratio

S.No.	Enzyme	Soret band (nm)	Delta band (nm)	Ratio (*S*/*D*)	Ref.
1	CYP101A1	409	351	1.4	58
2	T101V-CYP101A1	406	352	1.1	58
3	CYP102A1	410	350	2.2	59
4	P450_terp_	410	350	2.0	59
5	CYP175A1	408	350	1.22	This work
6	LFFTFA-CYP175A1	413	350	0.66	This work

## 3 Conclusions and future outlook

The present work demonstrates that the substrate scope of an enzyme can be expanded by rationally designing its active pocket, and the catalytic rate can be further increased by the engineering of various important channels such as the substrate entry channel and water channel. We have successfully developed a novel strategy for the rational evolution of enzymes based on computational tools. Computational data provided the rationale behind the absence of catalytic activity for hexadecane hydroxylation by wild-type enzyme. It is widely known that interactions beyond the primary coordination sphere also play a vital role in the enzymatic activity.^60^ Various reports have demonstrated the role of the second coordination sphere^61–63^ but it remains difficult to rationalise the factors responsible for modulating the activities of natural as well as artificial metalloenzymes. Our findings provide an in depth understanding and rationale to the role of these weak nonbonding interactions in the activity of the enzyme. An unfavorable environment inside the active pocket possibly due to the presence of polar residues (Q67 and Y68) was not allowing the binding of the non-polar substrate. Mutations of these polar residues with non-polar residues have shown the binding of the substrate, as evident from the detectable product formation. We found out that due to the presence of the bottleneck at the substrate entry channel (due to the bulky amino acids), the active site was not easily accessible by the substrate. Tunnel analysis using CAVER has shown that the replacement of bulky amino acids at the substrate entry channel (W269 and I270) with small amino acids can open up the bottleneck. An increase in the catalytic rate after performing W269F/I270A mutations confirms that the bottleneck has opened up. The residues near the substrate entry channel influencing the catalytic activity and selectivity of the enzyme have been previously reported, and the results agree well with our findings.^64^ The peroxide shunt pathway is known to be an inefficient method for cytochrome P450 catalysis. We envisioned that the catalytic rate through the peroxide shunt pathway can be increased by engineering the water channel in CYP175A1. To the best of our knowledge, there are only very few reports which could demonstrate that water channel engineering can lead to a higher catalytic rate through the peroxide shunt pathway. The mutation of non-polar residue V220 with the polar residue threonine, coupled with the mutation of L80 with phenylalanine has shown an increase in the catalytic rate by the shunt pathway. The thermal and thermodynamic stability of all the mutants were analyzed using CD spectroscopy. The results showed that the evolved enzymes were well folded and fairly thermally stable and suitable for working under ambient conditions. The secondary structure of the evolved enzymes remains unchanged but there was a significant change in the active site structure and polarity as evident from the CD data in the visible region. The present studies of structure-based rational design of the CYP175A1 enzyme can also be used for other enzymatic systems to expand the substrate scope and to increase the catalytic rate.

## Data availability

All relevant supporting data are provided in the ESI.†

## Author contributions

MT, KDD and SM conceptualized the problem, MT carried out all experimental work and part of the computational work, KDD carried out computational work, MT, KDD and SM interpreted the results and wrote the manuscript.

## Conflicts of interest

The authors declare no conflict of interest.

## Supplementary Material

SC-014-D3SC02857G-s001

SC-014-D3SC02857G-s002

SC-014-D3SC02857G-s003

SC-014-D3SC02857G-s004
