# Supplementary material for: Computationally guided bioengineering of the active site, substrate access pathway, and water channels of thermostable cytochrome P450, CYP175A1, for catalyzing the alkane hydroxylation reaction
Source: Chem Sci. 2023 Dec 4;14(48):14316–26. doi: 10.1039/d3sc02857g (PMC10718072; doi:10.1039/d3sc02857g)
Supplement: SC-014-D3SC02857G-s001 [file SC-014-D3SC02857G-s001.pdf]

Electronic Supplementary Material (ESI) for Chemical Science.
This journal is © The Royal Society of Chemistry 2023

Supporting Information

Computationally guided bioengineering of active site, substrate access pathway, and water channels of thermostable cytochrome P450, CYP175A1 for catalyzing alkane hydroxylation reaction

Mohd Taher[a*], Kshatresh Dutta Dubey[b*], Shyamalava Mazumdar[a*]

[a]Department of Chemical Sciences, Tata Institute of Fundamental Research, Homi Bhabha Road, Colaba, Mumbai 400005, India

[b]Department of Chemistry and Center for Informatics, School of Natural Science, Shiv Nadar University Delhi-NCR, NH91, Tehsil Dadri, Greater Noida, Uttar Pradesh 201314, India

correspondence: MT: chiraltaher@gmail.com, KDD: Kshatresh.dubey@snu.edu.in,

SM: shyamal@tifr.res.in

Running Title:Computationally guided bioengineering of tunnels and channels of a thermostable cytochrome P450 CYP175A1

Keywords: Computational enzyme design, Cytochrome P450, C-H activation, enzyme catalysis, Protein engineering.

Table of Content:

1. Materials …..(3)
2. Bacterial Strain and plasmids …..(3)
3. Expression of wild type and mutants of CYP175A1 …..(3)
4. Crystal structure superimposition of CYP175A1 with CYP102A1 …..(4)
5. Active site cavity detection using pymol …..(4)
6. Molecular Docking Protocol …..(5)
7. Definition of ligand binding site in WT and mutants of CYP175A1 …..(6)
8. Virtual Library of double mutant …..(7)
9. Hexadecane binding pose in mutants of CYP175A1 …..(9)
10. MD simulation protocol ......(10)
11. QM/MM protocol …..(11)
12. QM/MM optimised geometry coordinates …..(14)
13. Protein purification protocol ......(19)
14. PCR reaction protocol …..(19)
15. List of primers used for site directed mutagenesis …..(20)
16. Plasmid sequencing data …..(21)
17. CO Binding spectra …..(22)
18. Catalytic reaction condition …..(20)
19. Control experiments …..(23)
20. GC-FID analysis …..(23)
21. Determination of substrate consumption and product yield …..(24)
22. Calibration curve for substrate consumption …..(25)
23. Product analysis of the catalytic reaction by GC …..(25)
24. CD measurements …..(27)
25. Supporting video 1 (VS1) …..(31)
26. Supporting video 2 (VS2) …..(31)
27. Supporting video 3 (VS3) …..(31)
28. References …..(32)

1. Materials:

Component of bacteriological media (Tryptone, Yeast extract, Sodium chloride salt, Agar) were purchased from Himedia India. Isopropyl-β-D-1-thiogalactopyranoside (IPTG) was purchased from Bio Basic Canada Inc. Ni-NTA beads and plasmid isolation kit were purchased from QIAGEN. Ampicillin, Kanamycin, Chloramphenicol, hexadecane, octadecane, β Nicotinamide adenine dinucleotide, reduced dipotassium salt (NADPH), Lysozyme (from chicken egg white) and 4-amino luvolinic acid were purchased from Sigma-Aldrich. Hydrogen peroxide (H2O2) was purchased from SRL India. GC-MS grade solvents like ethyl acetate, chloroform and hexane were purchased from Merck. All the chemicals were used as supplied without any further purification until unless stated.

2. Bacterial Strain and plasmids:

E. coli XL10 Gold cells from agilent were used for plasmid DNA amplification and isolation. *E.coli* BL21(DE3) *codon plus* RP cells from agilent were used for protein expression. pRSFDuet-1 encoding for gene CYP175A1 from *Thermus thermophilus* HB27 was previously cloned and used.

3. Expression of wild type and mutants of CYP175A1:

BL21-DE3 *codon plus* RP cells were transformed with the expression plasmid pRSFDuet-1 encoding gene for CYP175A1. The transformed cells were grown overnight in 5 mL LB medium containing 34μg/mL chloramphenicol and 50 μg/mL kanamycin at 37 °C 200 rpm. The overnight grown culture was inoculated in 5 litter LB medium containing 34μg/mL chloramphenicol and 50μg/mL kanamycin and incubated at 37 °C 200rpm till the $O.D._{600}$ reached 0.6.-0.8. Expression of proteins were induced by adding 1mM IPTG and the culture was grown at 37 °C 200rpm for 16 hours. The cells were harvested by centrifuging at 5000rpm, 4 °C for 45 minutes. Pellet was stored at -20 °C refrigerator.

4. Crystal structure superimposition of CYP175A1 with CYP102A1

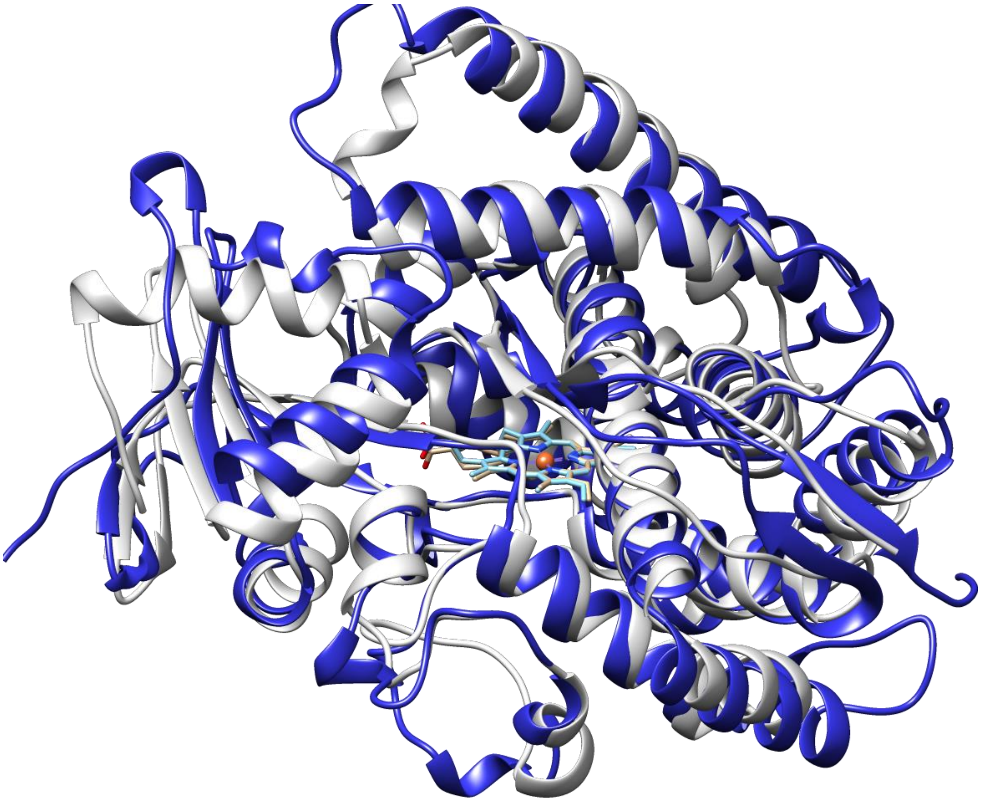

Figure S1: Superimposition of crystal structure of CYP175A1 (light grey) and CYP102A1 (blue).

5. Active site cavity detection using pymol

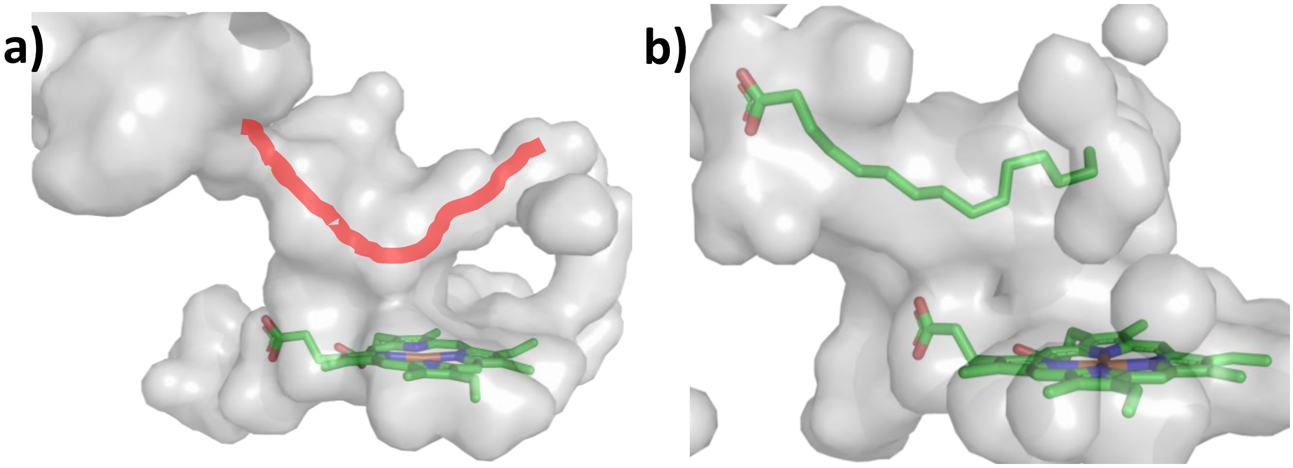

Figure S2: a) active site cavity of the wild-type CYP175A1 (PDB ID 1N97) showing a "U" type conformation (shape modelled in red colour for representation) b) Active site cavity of CYP102A1 (PDB ID 1FAG) showing the substrate bounds in the cavity.

6. Molecular Docking Protocol:

All the docking simulations were performed using protein crystal structure of CYP175A1 obtained from the protein databank entry code 1N97. Before starting docking calculations we have removed all the undesired residues like water, sulphate ions, ethylene glycol etc. from the crystal structure whereas additionally we have added all the hydrogen atoms in the protein. No hydrogen was added to sulphur atom of cys336 and kept as negatively charged. Mutations in the protein chain were made in Chimera using Dunbrack backbone-dependent rotamer library. Docking was performed using GOLD[1] (Genetic Optimization for Ligand Docking), version 5.3.0, in combination with the Chemscore scoring function[2] parameterized for heme containing proteins. All the docking poses were rescored using ChemPLP[3] scoring function to be ensure the reliability of results. The substrate binding site for docking was defined as the cavity in the distal pocket of the enzyme. The radius from this point was set to 1.5 nm to include the substrate access channel in the accessible volume for the docking. At most, 1000000 operations in the genetic algorithm were performed using a population of 500 genes. At least five independent docking simulations were performed to reach statistical significance. At least 10 poses were stored from every docking simulation, except if the best three docking poses had root-mean-square deviations smaller than 0.15 nm. To prevent an early convergence, a relative pressure of 1.1 was specified, and to account for diversity, the number of niches was set to 2. Ligand flexibility was specified as follows: The flipping of the free corners of ligand rings (if any), the flipping of amide bonds, and the flipping of planar nitrogen were allowed. Intramolecular hydrogen bonds were also allowed. To check the reliability of our docking methodology we performed control docking using CYP102A1and palmitoleic acid as well as CYP101 and 1s-Camphor. Docking results were very much similar to the crystal structure of the crystal structure of palmitoleic acid bound to CYP102A1 (PDB ID. 1FAG). Highest scoring pose had a RMSD of 0.2 Å with the experimental enzyme substrate complex (1FAG). In case of CYP101 substrate bound complex (2CPP) and camphor docking results were even more reliable with the RMSD of 0.01 Å.

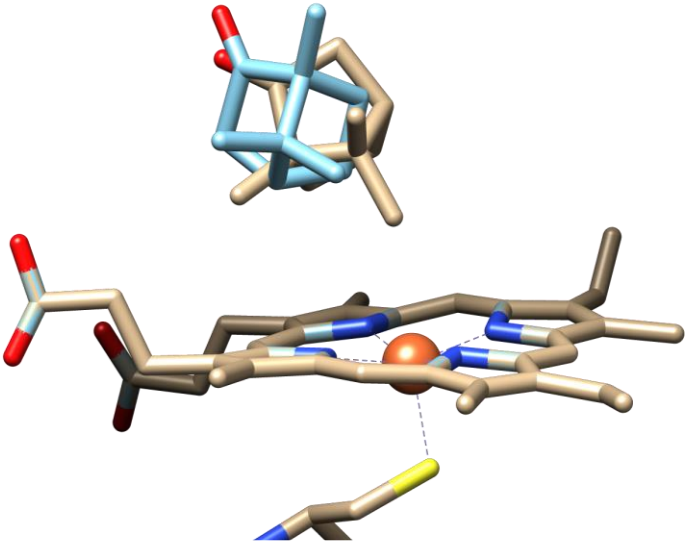

Figure S3: Control docking experiment performed on CYP101A1 (cyan colour represents docked camphor and golden colour represents crystal structure of camphor).

7. Definition of ligand binding site in WT and mutants of CYP175A1:

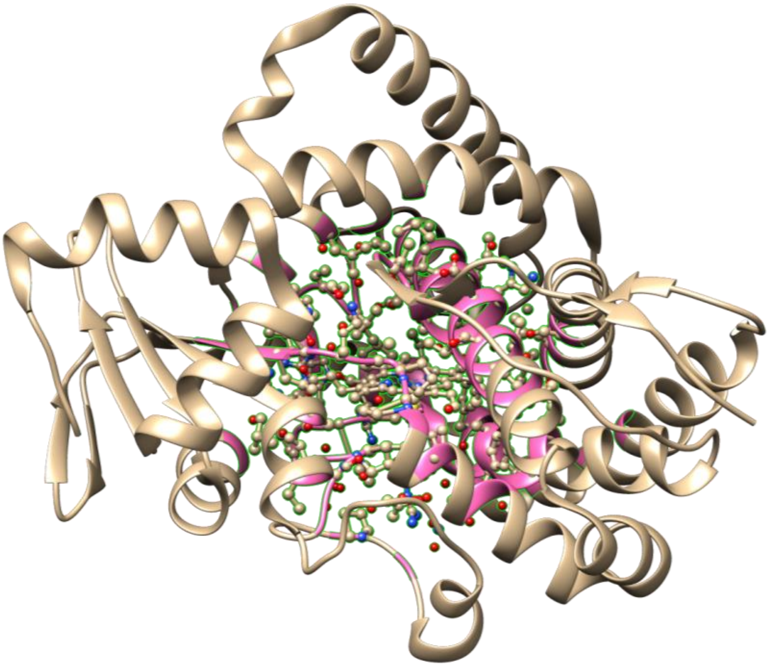

Figure S4: Highlighted residues represents the binding site defined in the crystal structure of wild type CYP175A1 for the docking studies.

Polar	Non-Polar
Q67	L71
Y68	L80
E224	L161
T225	V220
T372	A221
	A268
	I270
	L271
	V371

Table S1: Amino acids present with in 5Å distance from the hexadecane from the highest scoring docking pose categorised according to their polarity

8. Virtual Library of double mutant:

S.No.	CYP175A1 Variant
1	Wild Type
2	Q67A_Y68A
3	Q67I_Y68I
4	Q67L_Y68L
5	Q67F_Y68F
6	Q67L_Y68F
7	Q67V_Y68V
8	Q67A_Y68V
9	Q67I_Y68V
10	Q67L_Y68V
11	Q67F_Y68L
12	Q67M_Y69M
13	Q67W_Y68Y
14	Q67W_Y68W

Table S2: Virtual library of double mutant of CYP175A1. Q67 and Y68 residues have been replaced by the non-polar amino acids.

S.No.	CYP175A1 Variant	Ligand	Docking Score*	Ligand binding Pose#
1	Wild Type	Hexadecane	35.32	Nearest atom of Hexadecane is C1 and have a C1-Fe (Heme) distance of 3.28Å.
2	Q67A_Y68A	Hexadecane	36.59	Nearest atom of Hexadecane is C1 and have a C1-Fe (Heme) distance of 3.27Å.
3	Q67I_Y68I	Hexadecane	38.25	Nearest atom of Hexadecane is C3 and have a C3-Fe (Heme) distance of 3.31Å.
4	Q67L_Y68L	Hexadecane	37.21	Nearest atom of Hexadecane is C1 and have a C1-Fe (Heme) distance of 3.26Å.
5	Q67F_Y68F	Hexadecane	40.56	Nearest atom of Hexadecane is C8 and have a C8-Fe (Heme) distance of 3.78Å
6	Q67L_Y68F	Hexadecane	41.01	Nearest atom of Hexadecane is C6 and have a C6-Fe (Heme) distance of 3.75Å
7	Q67V_Y68V	Hexadecane	36.97	Nearest atom of Hexadecane is C1 and have a C1-Fe (Heme) distance of 3.45Å.
8	Q67A_Y68V	Hexadecane	37.36	Nearest atom of Hexadecane is C2 and have a C2-Fe (Heme) distance of 3.07Å.
9	Q67I_Y68V	Hexadecane	37.54	Nearest atom of Hexadecane is C1 and have a C1-Fe (Heme) distance of 3.37Å.
10	Q67L_Y68V	Hexadecane	36.74	Nearest atom of Hexadecane is C3 and have a C3-Fe (Heme) distance of 3.48Å.
11	Q67F_Y68L	Hexadecane	39.83	Nearest atom of Hexadecane is C2 and have a C2-Fe (Heme) distance of 3.34Å.
12	Q67M_Y69M	Hexadecane	37.74	Nearest atom of Hexadecane is C2 and have a C2-Fe (Heme) distance of 3.13Å.
13	Q67W_Y68Y	Hexadecane	43.32	Nearest atom of Hexadecane is C4 and have a C4-Fe (Heme) distance of 4.57Å.
14	Q67W_Y68W	Hexadecane	47.15	Nearest atom of Hexadecane is C5 and have a C5-Fe (Heme) distance of 5.30Å.
**S.No.**	**CYP101A1**	**Ligand**	**Docking Score***	**Ligand binding Pose#**
15	P450Cam	1s-Camphor	30.95	RMSD with the crystal structure is 0.01Å.

Table S3: Results of hexadecane docking in the virtual library of double mutant of CYP175A1. *docking score represent the Chem score scoring function[1]. #docking pose in the lowest energy docked complex.

9. Hexadecane binding pose in mutants of CYP175A1

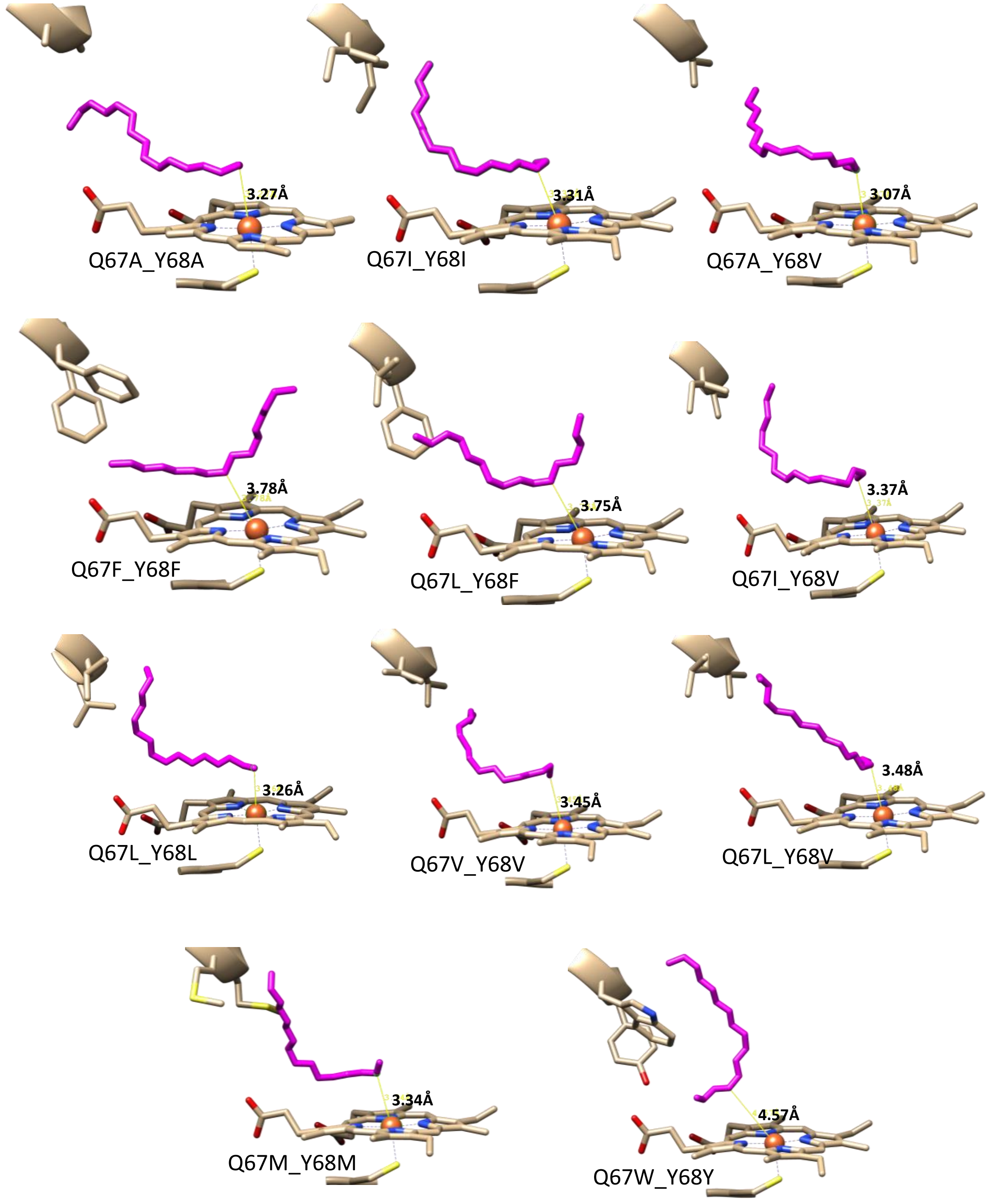

Figure S5. Hexadecane (maroon colour) binding conformation in the binding site of various mutants of CYP175A1. Data showing the pose of highest scoring docking solution. Figures were made in chimera.

10. MD Simulations Protocol:

The best-docked poses were selected for the MD Simulations. The missing hydrogen atoms of the enzyme (PDB id: 1N97) were added using the Leap Module of the Amber 20 MD package using the Amber ff19SB force field[4]. The heme parameters for the heme were developed by Cheatham et al, compatible with the Amber forcefiled and we used the same in our study[5]. Parameters for the substrate, hexadecane, were prepared using an antechamber module of the Amber MD package using Generalized Amber Force Field 2 (GAFF2). Partial charges of the substrate were calculated using the RESP (restrained electrostatic potential) method[6] of a QM optimized geometry at HF/6-31 G (d, p) level of theory. After system parameterization, the enzyme-substrate complex was soaked into a TIP3P water box extending up to 12Å from the protein boundary. An appropriate number of counter ions were added to neutralize the charge of the system.

After proper system setup, the complex was minimized in two steps; in the first step solvent minimization was performed and in the second step entire system was minimized without any restraint using 5000 steps of steepest descent followed by 5000 steps of conjugates gradient algorithm. The system was then gently heated from 0 to 300 K for 50 ps using the NVT ensemble, followed by 1 ns simulation at the NPT ensemble at 300 K and 1.0 atm using the Langevin thermostat[7] and Berendsen barostat[8] with a collision frequency of 2 ps and a pressure relaxation time of 1 ps. The equilibrated system was further subjected to a further productive run for 100ns. The Monte Carlo barostat was used during all production MD simulations. Moreover, replica simulations were performed to ensure the consistency of the obtained results. The SHAKE algorithm[8] was employed to constrain the hydrogen bonds, while particle mesh Ewald (PME)[9] and appropriate cutoff distances (12 Å) were used to treat the long-range electrostatic and van der Waals forces, respectively. All MD simulations were carried out in the GPU version of the AMBER20 package[10]. The CPPTRAJ module of the AMBER 20 was used to analyze all the results. A similar protocol was used for all mutants of the CYP175A1 enzyme.

11. QM/MM Calculations protocol:

The C-H activation mechanism was investigated using QM/MM calculations. QM-region includes heme porphyrin with ligated cysteine, substrate, and a water molecule. For cysteine, we used S-H and therefore we cut the S-C bond as QM/MM interface. All protein residues and water molecules within 8Å of the heme were included in the active region of the QM/MM calculations. The atoms in the active region interact with the QM atoms through electrostatic and van der Waals interactions and the corresponding polarization effects were considered in the subsequent QM energy. All QM/MM calculations were performed with ChemShell[11] , by combining Turbomole[12] for the QM part, and DL_POLY for the MM part. The MM region was treated using the Amber ff19SB force field and the electronic embedding scheme was used to account for the polarizing effect of the enzyme environment on the QM region. The QM/MM boundary was treated using hydrogen link atoms with the charge shift model[13] (10). During QM/MM geometry optimizations and frequency calculations, the QM region was treated using the hybrid UB3LYP functional[14] with a def2-SVP basis set. All of the QM/MM transition states were located by relaxed potential energy surface (PES) scans followed by full TS optimizations using the P-RFO optimizer implemented in the HDLC code. The zero-point energies (ZPE) for all the species were further corrected and values were added with dispersion correction[15] . The final energy was further corrected by single-point energy calculations with a higher basis set at UB3LYP/def2-TZVP+ZPE level of theory. The basis set and QM method were chosen on the basis of several similar previous studies in P450 chemistry.[16] The Cpd I (RC) mediated P450 reactions can be observed in two different spin states that is doublet and quartet. It is noteworthy that our calculations suggest that the reaction will not proceed through the quartet spin state since the barrier is higher compared to that in the doublet state. , therefore all results were taken from the doublet states, which is the favoured one. However, the calculations for the quartet spin state have been added in Figure S6 for comparison.

**Species**	**^{2}RC**	**^{2}IM**	**PC**
Fe	1.25799	2.05723	1.27011
O	0.86793	0.16375	0
S	-0.16518	-0.08968	-0.09156
Porphyrin	-0.997	-0.17	-0.22
Substrate	0	-0.96	0

Table S4: Spin densities of the stationary points included in the QM/MM calculations for the doublet spin states. Calculations were done using B3LYP/def2-TZVP level of theory.

**Species**	**^{4}RC**	**^{4}IM**	**PC**
Fe	1.16045	2.09752	2.6899
O	0.88772	0.15982	0
S	0.12909	-0.08580	0.33071
Porphyrin	0.86978	-0.18037	-0.10065
Substrate	0	0.96556	0

Table S5: Spin densities of the stationary points included in the QM/MM calculations for the quartet spin states. Calculations were done using B3LYP/def2-TZVP level of theory.

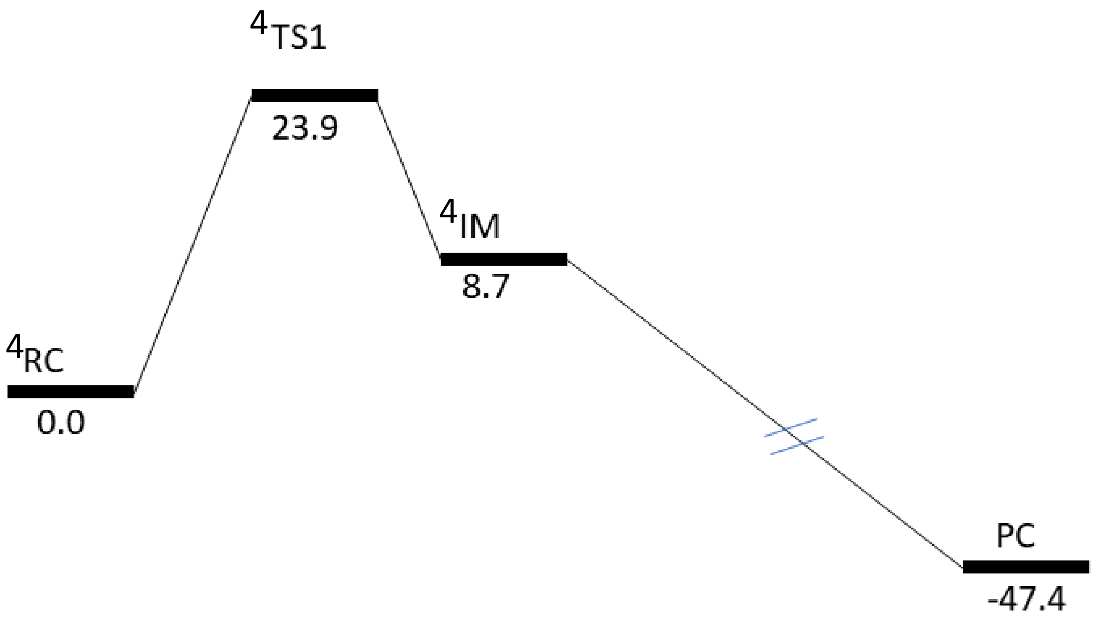

Figure S6: Energy profile in the Quartet state. Calculations were done using B3LYP/def2-TZVP level of theory. QM/MM energy of ^{2}RC (doublet) = 0.0 kcal/mol (reference energy for Fig 5b) QM/MM energy of ^{4}RC (quartet) =6.1 kcal/mol. (all energy values are in kcal/mol)

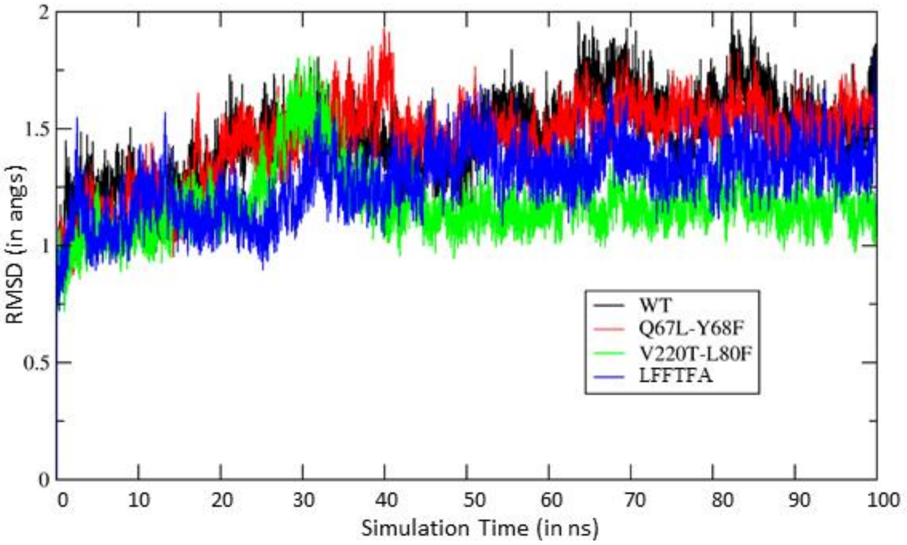

Figure S7: RMSD (in Å) for protein backbone od the wild-type and mutants of CYP175A1 during MD simulations.

12. QM/MM Optimized Geometry:

Provided geometry are for truncated QM zone.

RC

C	41.4835476	39.6602241	36.9267651
H	40.3966598	39.8288425	37.0419805
C	42.1928012	40.5756367	37.9243420
H	41.8509556	40.3841222	38.9556620
H	43.2877706	40.4459451	37.9046873
H	41.9835933	41.6315440	37.6891132
O	41.8016664	39.9527224	35.5761601
H	42.6804611	40.3923752	35.5284728
S	39.1764645	46.0609709	34.7475189
N	41.6598994	44.3894718	34.6798041
C	41.3324438	43.2362274	34.0398898
C	42.6957146	44.9384101	33.9631947
C	42.1406259	43.0577065	32.8409425
C	43.0500165	44.0856431	32.8241861
C	43.2689562	46.1715584	34.2707282
H	44.0846948	46.5089654	33.6318727
C	42.9108753	47.0592454	35.2952424
N	41.9284162	46.8127036	36.2128220
C	41.9027930	47.9022540	37.0410458
C	42.8491353	48.9065007	36.5939908
C	43.5127565	48.3674091	35.5175060
C	41.1255913	47.9858444	38.1956210
H	41.2596504	48.8831229	38.8016263
C	40.1858549	47.0620030	38.6499298
C	39.3685689	47.2232171	39.8323680
C	38.8410718	45.3332084	38.7254550
N	39.8495114	45.9027199	38.0005415
C	38.5042659	46.1625812	39.8709127
C	38.2532202	44.0992949	38.4189137
H	37.4402566	43.7878955	39.0724419
C	38.5987116	43.2229974	37.3936034
C	37.9654649	41.9307678	37.1445352
N	39.5794394	43.4725444	36.4695125
C	39.5826803	42.3918167	35.6386298
C	40.4052825	42.2966601	34.5112100
H	40.3364197	41.3853832	33.9250920
Fe	40.8282083	45.0867302	36.4123549
C	38.5745220	41.3965434	36.0363097
O	41.9445048	44.4028357	37.3777839
C	45.8845421	43.2587478	40.6153127
C	44.7709801	42.2028204	40.5391205
C	43.3495577	42.7631261	40.7256364
C	43.1367194	43.6267920	41.9783471
C	43.4111934	42.9099017	43.3080514
H	45.9493756	43.6826563	41.6323997
H	44.9582379	41.4144008	41.2901142
H	45.6772577	44.0989218	39.9285085
H	42.6377350	41.9183892	40.7443921
H	44.8019718	41.6853779	39.5650175
H	42.1017394	44.0184346	41.9582674
H	43.0785187	43.3636470	39.8385891
H	44.4395769	42.5196636	43.3175868
H	43.7938583	44.5113996	41.8966288
H	42.7495900	42.0380302	43.4471675
H	41.6441103	38.6109547	37.1743949
H	38.2629077	46.8912852	35.3642355
H	41.9179526	42.3133489	32.0764718
H	43.9023293	44.2654064	32.1689646
H	42.9425521	49.9133635	37.0008910
H	44.3380009	48.8180917	34.9662129
H	39.4442262	48.0424406	40.5473617
H	37.7310140	46.0219726	40.6261463
H	37.2458934	41.4643377	37.8173851
H	38.4093478	40.4523013	35.5174731
H	46.8657952	42.8561826	40.3640146
H	43.2888562	43.5678793	44.1683805

12. QM/MM Optimized Geometry:

Provided geometry are for truncated QM zone**.**

IM

C	41.5031145	39.6667442	36.8887402
H	40.4188640	39.8480907	37.0097711
C	42.2295636	40.5875116	37.8697924
H	41.8867782	40.4144397	38.9046636
H	43.3220671	40.4387843	37.8501208
H	42.0419585	41.6425810	37.6119509
O	41.8163068	39.9389141	35.5333036
H	42.6934261	40.3828696	35.4752926
S	39.2035673	46.0572121	34.7494809
N	41.6514435	44.3771442	34.6280516
C	41.3312186	43.2152300	34.0047669
C	42.6720758	44.9279659	33.8953176
C	42.1341000	43.0272231	32.7998094
C	43.0286717	44.0659573	32.7612412
C	43.2391623	46.1665935	34.1938029
H	44.0401570	46.5136407	33.5408943
C	42.8990000	47.0444155	35.2356470
N	41.9345618	46.7851863	36.1653133
C	41.9249137	47.8621888	37.0105605
C	42.8652421	48.8729533	36.5613864
C	43.5094462	48.3479724	35.4662617
C	41.1697066	47.9302652	38.1810772
H	41.3127871	48.8244605	38.7900320
C	40.2376248	47.0029814	38.6528420
C	39.4309855	47.1703828	39.8421928
C	38.8686321	45.2950728	38.7287493
N	39.8874567	45.8478868	38.0039832
C	38.5522833	46.1199484	39.8826407
C	38.2498521	44.0792226	38.4007352
H	37.4306136	43.7746977	39.0494041
C	38.5796335	43.2097811	37.3641519
C	37.9484979	41.9176448	37.1137685
N	39.5633683	43.4613544	36.4412486
C	39.5752453	42.3782921	35.6147725
C	40.4071566	42.2785219	34.4900847
H	40.3430665	41.3608275	33.9133281
Fe	40.7807665	45.0946895	36.3322190
C	38.5681042	41.3806888	36.0113710
O	42.0621091	44.2928943	37.3328036
C	45.8378519	43.2761917	40.6652684
C	44.6923275	42.2876771	40.3864664
C	43.3300010	42.7423123	40.8198284
C	43.1075715	43.6136962	42.0171098
C	43.3770189	42.9002147	43.3665610
H	45.9221705	43.4820895	41.7460854
H	44.9197150	41.3142806	40.8779117
H	45.6476752	44.2394909	40.1615700
H	42.4729302	42.1947859	40.4117684
H	44.6495901	42.0383149	39.3118939
H	42.0758596	44.0109736	41.9998455
H	41.7353769	44.1112330	38.2281793
H	44.3940168	42.4815746	43.3599787
H	43.7803168	44.4892779	41.9451863
H	42.6929402	42.0499233	43.5195284
H	41.6554456	38.6190317	37.1478944
H	38.2912958	46.8750656	35.3844799
H	41.9149843	42.2697705	32.0472663
H	43.8737373	44.2505337	32.0980196
H	42.9649948	49.8755013	36.9773359
H	44.3305168	48.8008043	34.9105157
H	39.5113291	47.9927172	40.5530924
H	37.7819335	45.9883309	40.6424467
H	37.2235009	41.4521560	37.7814261
H	38.4104729	40.4323617	35.4976636
H	46.8053787	42.9015698	40.3311763
H	43.2833166	43.5740374	44.2181772

12. QM/MM Optimized Geometry:

Provided geometry are for truncated QM zone**.**

TS1

C	41.5611941	39.5655607	36.8294041
H	40.4872536	39.7795276	36.9799188
C	42.3425756	40.4748441	37.7711892
H	42.0474651	40.3195603	38.8227003
H	43.4304743	40.3104254	37.7014312
H	42.1524824	41.5279824	37.5140806
O	41.8481748	39.8151038	35.4630203
H	42.7241252	40.2519559	35.3783600
S	39.3306990	46.0660813	34.9219163
N	41.7908949	44.4327993	34.8366454
C	41.4528217	43.2878924	34.1897494
C	42.7882238	45.0066520	34.0864959
C	42.2254251	43.1305172	32.9622166
C	43.1198171	44.1696172	32.9268075
C	43.3437272	46.2496614	34.3834480
H	44.1350746	46.6050573	33.7240083
C	42.9874346	47.1293107	35.4170608
N	42.0280882	46.8624678	36.3512815
C	41.9788502	47.9595299	37.1701326
C	42.8905072	48.9854068	36.7000443
C	43.5560351	48.4552348	35.6204781
C	41.2064493	48.0380264	38.3277296
H	41.3126246	48.9535167	38.9121006
C	40.2935461	47.0966510	38.8076843
C	39.4420207	47.2835462	39.9628417
C	38.9483923	45.3689238	38.8828215
N	39.9886095	45.9159790	38.1843828
C	38.5735058	46.2250758	39.9966363
C	38.3546080	44.1404495	38.5642312
H	37.5104280	43.8453918	39.1856569
C	38.7192810	43.2565355	37.5504705
C	38.0675367	41.9799132	37.2752831
N	39.7218142	43.4992689	36.6468418
C	39.7073549	42.4348340	35.7918762
C	40.5262930	42.3477886	34.6605320
H	40.4475967	41.4432393	34.0652318
Fe	40.9489591	45.1287549	36.5720904
C	38.6719574	41.4552428	36.1589124
O	42.2501092	44.4424106	37.5309516
C	45.1186661	43.7457305	39.9977966
C	43.9294362	42.8251421	39.6705420
C	42.5281660	43.4205370	39.8231913
C	42.2652428	44.2021866	41.1041544
C	42.5028497	43.3635791	42.3800439
H	45.0345916	44.1624356	41.0159474
H	43.9744225	41.9185665	40.3064435
H	45.1576356	44.5992973	39.3002617
H	41.7734688	42.6259063	39.6984373
H	44.0168776	42.4492833	38.6413162
H	41.2389886	44.6092398	41.0838256
H	42.3178133	44.0824783	38.7792830
H	43.5549917	43.0461948	42.4315261
H	42.9379072	45.0715802	41.1412328
H	41.9041098	42.4394289	42.3675391
H	41.6894283	38.5184055	37.1034279
H	38.3942301	46.8823436	35.5228232
H	41.9828217	42.3909674	32.1991459
H	43.9485594	44.3679128	32.2471515
H	42.9643329	50.0004299	37.0903583
H	44.3587132	48.9327327	35.0585432
H	39.4978704	48.1168143	40.6632789
H	37.7633189	46.1123440	40.7170222
H	37.3399337	41.5148536	37.9404000
H	38.4811214	40.5310718	35.6134248
H	46.0683564	43.2129742	39.9495656
H	42.2757050	43.9125346	43.2938931

12. QM/MM Optimized Geometry:

Provided geometry are for truncated QM zone**.**

TS2

```
C   41.5846832  39.5938555  36.8163099
H   40.5168520  39.8203630  36.9885166
C   42.3926285  40.5111639  37.7315302
H   42.1126896  40.3745463  38.7902706
H   43.4788059  40.3401823  37.6462607
H   42.2029832  41.5603047  37.4573432
O   41.8428694  39.8240935  35.4411588
H   42.7199077  40.2542313  35.3284503
S   39.2603285  46.1237317  34.8257479
N   41.7235126  44.5506348  34.8227147
C   41.4083885  43.3975242  34.1832850
C   42.7316947  45.1262111  34.0814406
C   42.2019307  43.2306865  32.9700586
C   43.0864539  44.2778349  32.9343067
C   43.2831922  46.3694694  34.3774704
H   44.0835786  46.7214980  33.7260932
C   42.9149657  47.2674192  35.3965052
N   41.9415027  47.0206050  36.3207964
C   41.8687163  48.1448906  37.1010396
C   42.7936833  49.1573211  36.6279052
C   43.4824504  48.5969627  35.5768397
C   41.0649822  48.2418976  38.2389221
H   41.1471199  49.1696607  38.8088734
C   40.1605612  47.2945876  38.7214817
C   39.3340313  47.4576286  39.8985776
C   38.8757187  45.5234022  38.8352505
N   39.8728011  46.1026975  38.1041227
C   38.5022444  46.3706184  39.9576546
C   38.3281973  44.2621229  38.5545492
H   37.5048360  43.9526758  39.1965329
C   38.7093981  43.3626021  37.5601619
C   38.0811725  42.0642368  37.3169459
N   39.6973161  43.6117495  36.6429277
C   39.6845873  42.5338337  35.7997081
C   40.4889529  42.4505794  34.6611355
H   40.4211901  41.5387253  34.0745361
Fe  40.8362488  45.2811133  36.5214056
C   38.6740107  41.5372051  36.1975625
O   42.2092411  44.6172825  37.5869837
C   45.2100118  43.9731316  39.8897300
C   44.0404324  43.1180726  39.3704495
C   42.6549855  43.5580170  39.7169970
C   42.3681700  44.3575935  40.9477886
C   42.5527051  43.5068687  42.2362784
H   45.1267874  44.1462921  40.9753702
H   44.1447536  42.0719158  39.7338330
H   45.2211036  44.9630088  39.4028880
H   41.8509310  42.9258915  39.3376500
H   44.0708039  43.0248758  38.2742174
H   41.3454652  44.7689415  40.9008849
H   42.7249657  45.3661767  37.9205808
H   43.5876256  43.1381981  42.2960469
H   43.0666978  45.2085094  41.0028225
H   41.9061362  42.6164677  42.2129666
H   41.7085540  38.5485583  37.0992884
H   38.3097113  46.9390323  35.4053678
H   41.9817016  42.4744062  32.2166620
H   43.9212893  44.4757814  32.2620463
H   42.8569611  50.1789467  37.0025306
H   44.3030799  49.0365691  35.0099353
H   39.4025352  48.2798251  40.6108748
H   37.7277852  46.2207097  40.7098563
H   37.3755291  41.5946175  38.0022139
H   38.4916305  40.6046071  35.6636472
H   46.1747943  43.4946725  39.7213889
H   42.3379472  44.0693569  43.1448780
```

12. QM/MM Optimized Geometry:

Provided geometry are for truncated QM zone**.**

PC (product)

C	41.4693268	39.6007794	36.9446837
H	40.3842102	39.7716209	37.0710582
C	42.1851038	40.5155010	37.9381168
H	41.8526628	40.3270720	38.9728367
H	43.2806645	40.3913301	37.9090715
H	41.9696264	41.5695666	37.7009888
O	41.7788384	39.8913596	35.5940086
H	42.6541615	40.3307766	35.5396057
S	39.0898480	46.0668215	34.6861242
N	41.5806649	44.4903556	34.6190432
C	41.2545361	43.3226188	33.9807510
C	42.6129086	45.0319171	33.8866913
C	42.0668477	43.1342117	32.7883853
C	42.9728065	44.1635702	32.7619422
C	43.2054091	46.2554945	34.1869463
H	44.0047062	46.6010582	33.5323893
C	42.8806459	47.1096889	35.2387766
N	41.9169667	46.8487785	36.1936337
C	41.9029747	47.9588494	37.0136456
C	42.8468023	48.9514035	36.5473260
C	43.4946798	48.4066717	35.4631119
C	41.1337166	48.0737292	38.1658480
H	41.2618765	48.9819343	38.7558217
C	40.2065997	47.1461523	38.6229556
C	39.4183951	47.2922196	39.8266483
C	38.8649326	45.4083282	38.7197776
N	39.8672722	45.9826881	37.9675613
C	38.5569634	46.2308154	39.8763398
C	38.2881168	44.1753364	38.4385298
H	37.4873135	43.8529403	39.1008143
C	38.6488542	43.3027027	37.4162635
C	38.0454584	41.9968900	37.1983898
N	39.6135060	43.5650045	36.4673741
C	39.5876752	42.4739527	35.6218659
C	40.3488096	42.3840567	34.4623293
H	40.2700187	41.4697896	33.8828088
Fe	40.5803159	45.3132451	36.2029363
C	38.6201149	41.4689428	36.0666256
O	43.2735972	43.6486945	39.3557531
C	45.9480943	43.3562104	40.5337690
C	44.8041573	42.3438353	40.3964477
C	43.3885224	43.0728117	40.2390141
C	43.0609674	43.8586216	41.6068230
C	43.1353213	43.0525071	42.9034155
H	45.9577728	43.8047262	41.5413927
H	44.8010549	41.6384498	41.2406633
H	45.8319879	44.1962827	39.8158357
H	42.6693963	42.2032526	40.3021072
H	44.9297553	41.7344771	39.4824949
H	42.0517793	44.2910021	41.4521951
H	43.9586258	44.3057133	39.1343704
H	44.1704523	42.7327356	43.0946761
H	43.7547250	44.7236028	41.6518852
H	42.5351099	42.1283389	42.8385855
H	41.6315137	38.5522885	37.1945424
H	38.2209826	46.9073587	35.3516447
H	41.8608281	42.3717854	32.0371668
H	43.8321098	44.3312077	32.1126636
H	42.9435869	49.9627924	36.9420316
H	44.3249441	48.8423528	34.9073149
H	39.5161015	48.0992675	40.5527298
H	37.8040874	46.0801517	40.6499969
H	37.3765825	41.5110306	37.9087508
H	38.4476675	40.5182011	35.5622257
H	46.9336484	42.9330835	40.3395682
H	42.7893063	43.6133329	43.7716408

13. Protein purification protocol:

The cell pellet was taken out from -20 °C and re- suspended in lysis buffer (10 mL of 50mM tris with 150mM NaCl, 1%Triton X100, 1mg/mL Lysozyme, 1mM EDTA, 1mM AEBSF- for pellet of 2 litter culture) and kept on rotary at 4 °C for 1 hour. The cell lysate was disrupted by sonication with 40% amplitude for 1 hour with 30 sec on - 30 sec off on ice bath. The cell debris was removed by centrifugation at 17000rpm, 4 °C for 60 minutes. The supernatant was poured into 7 mL equilibrated Ni-NTA beads. Kept beads on rotary for 10-12 hours at 4 °C. The beads were then poured into Ni-NTA affinity column and flow through was collected in a 50 mL falcon. Beads were washed with 40 mL binding buffer (50 mM Tris, 150 mM NaCl, 1% Glycerol pH8). Again beads were further washed with 30mL wash1 buffer containing binding buffer with 30mM imidazole. Further washed beads with 30mL wash2 buffer containing binding buffer with 50mM imidazole. Finally protein was eluted in 15 mL elution buffer containing binding buffer with 250mM imidazole. Purified protein solution was concentrated using 10 KDa membrane filter by centrifuging 2-3 times at 3500 rpm for 10 minutes. Protein was further purified by size exclusion chromatography using Superdex 75PG column.

14. PCR reaction protocol:

The site directed mutagenesis of the CYP175A1 gene were carried out using Platinum Superfi DNA polymerase PCR kit from Invitrogen. The plasmid was isolated from the freshly transformed XL10-Gold *E.coli* cells and the purity was checked by running agarose gel electrophoresis (1% agarose). Ethidium Bromide was used for staining and 1kb DNA ladder (from Invitrogen) was used as marker. The purified plasmid was used to carry out the polymerase chain reactions to amplify the structural gene with the introduction of point mutation. PCR reaction and the thermal cycling parameters were used as per the protocol supplied by the mutagenesis kit. The list of primers used for making mutant of CYP175A1 are listed below.

15. List of primers used for site directed mutagenesis:

W269F_I270A Fw: 5’- TACCCGCCGGCATTCGCTCTGACCCGTC -3’

W269F_I270A Rev: 5’- GACGGGTCAGAGCGAATGCCGGCGGGTA -3’

V220T_Fw: 5’-GGTGACGCTGCTGACTGCAGGTCATGAAAC-3’

V220T_Rev: 5’-GTTTCATGACCTGCAGTCAGCAGCGTCACC-3’

L80F_Fw: 5’-CGGTCGTGGTCTGTTCACCGATTGGGGCGA-3’

L80F_Rev: 5’-TCGCCCCAATCGGTGAACAGACCACGACCG-3’

Q67L_Y68F Fw:5’-GCCACCTTCCTGTTTCGTGCACTG-3’

Q67L_Y68F Rev:5’-CAGTGCACGAAACAGGAAGGTGGC-3’

Step	Temperature	Time (minutes)
**Initial denaturation**	**95 °C**	**5**
**Denaturation**	**95 °C**	**1** } 30 Cycles
**Annealing**	**55 °C- 58 °C**	**1** } 30 Cycles
**Extension**	**72 °C**	**6** } 30 Cycles
**Final extension**	**72 °C**	**15**

Table S6: PCR reaction thermal cycles parameters.

16. Plasmid sequencing data

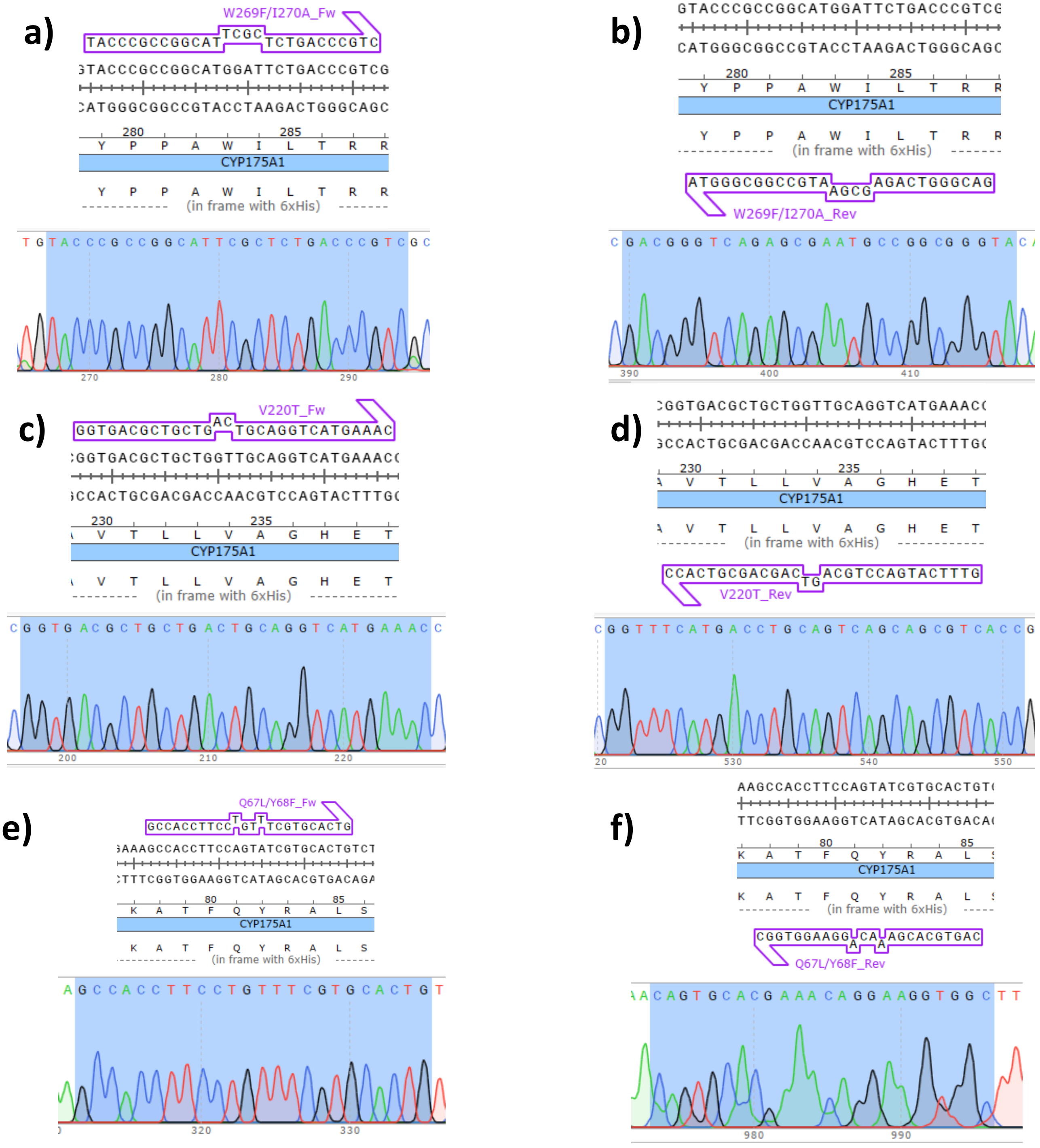

Figure S8: Raw data of Plasmid sequencing. A) Forward primer for the W269F/I270A mutation (above) and corresponding plasmid sequencing data (below). B) Reverse primer for the W269F/I270A mutation (above) and corresponding plasmid sequencing data (below). C) Forward primer for the V220T mutation (above) and corresponding plasmid sequencing data (below). D) Reverse primer for the V220T mutation (above) and corresponding plasmid sequencing data (below). E) Forward primer for the Q67L/Y68F mutation (above) and corresponding plasmid sequencing data (below). F) Forward primer for the Q67L/Y68F mutation (above) and corresponding plasmid sequencing data (below).

17. CO Binding spectra:

CO-binding spectrum (Figure S9) was recorded according to earlier published reports.[17] Briefly, Carbon monoxide was prepared by reacting sulphuric acid (H_2SO_4) with formic acid (HCOOH). Fumes of carbon monoxide were purged into CYP175A1 protein solution in a cuvette and few grains of sodium dithionite were added. This solution was then analysed for shifting of soret band to 450 nm by UV-VIS spectroscopy.

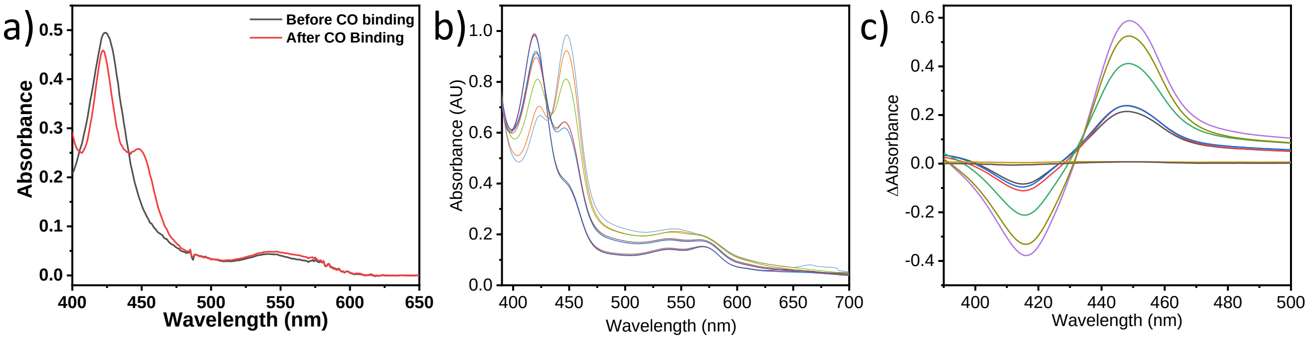

Figure S9: CO binding spectrum of LF and LFFTFA mutant of CYP175A1. a) CO binding spectrum of LF mutant showing characteristic peak shift from 417 nm to 450nm. b) CO binding spectrum of LFFTFA mutant showing characteristic peak shift from 417 nm to 450nm. c) CO binding difference spectrum of LFFTFA mutant showing characteristic peak maxima at 450nm.

18. Catalytic reaction condition:

Reaction conditions for alkane hydroxylation were same of wild type and all the mutants of CYP175A1. In a typical reaction system 10 μM of enzyme was incubate with 1mM hexadecane solution for 20min. Monooxygenation reaction was started by adding 10mM H_2O_2. Stock solutions of the substrates was prepared using ethanol as solvent. Total reaction volume was 1ml was added in a 20ml screw cap scintillation vial and stirred with magnetic bead in it. Reaction vials were incubated at 50 °C with constant stirring and products were analysed after 24 hours using GC-MS and GC-FID. All the experiments were performed at least in triplicate. All the catalytic reactions were performed under the similar conditions until otherwise stated.

19. Control experiments:

Three sets of reactions as the control experiments were performed. In first case, enzyme (10 uM) was mixed with substrate (1mM) and the reaction mixture was incubated at 50 °C for 24 hours and the reaction was analysed using GC-MS and GC-FID. In the second set of control experiment, substrate (1mM) was mixed with H_2O_2 (10mM) and the mixture was incubated at 50 °C for 24 hours. In the third control experiment, enzyme (1uM) was mixed with H2O2 (10mM) and the mixture was incubated at 50 °C for 24 hours. All the control experiments were analysed by GC-MS and GC-FID.

20. GC-FID analysis:

All the reactions were analysed using gas chromatography instrument coupled with flame ionisation detector (GC-FID). Catalytic reactions were stopped at different time interval by adding 20µl of 2M HCl and 10uL of 0.4mM Octadecane solution (in acetonitrile) was added as internal standard. Reaction products were isolated by solvent extraction method using $CHCl_3$. $CHCl_3$ layer was taken and dried over anhydrous Na_2SO_4 and subjected to GC-MS analysis. All samples were injected at a volume of 1.0 µl, and analyses were performed at least in triplicate. All analysis were performed on Trace GC ultra instrument by Thermo electron corporation system having GsBP-5MS, 30m x 0.25mm x0.25µm column fitted into the machine. A typical GC temperature program for separating the reactant and products was 250°C PTV injector with split flow rate of 50ml/min, 50 °C oven for 2 min, then 15 °C/min gradient to 220 °C hold at 220 °C for 3min, 15 °C/min gradient to 300 °C, and then 300 °C for 6 min. FID parameters were as follows: Flame temperature was 250 °C, zero air= 350ml/min, hydrogen= 35ml/min, Nitrogen=30ml/min.

21. Determination of substrate consumption and product yield:

Calibration curve for area v/s concentration for the substrate (Hexadecane) and internal standard (octadecane) were plotted as shown in Figure S11 and the substrate consumption was determined by comparing the relative peak area ratios of substrate and internal standard. This substrate consumption is directly proportional to product formation and was used for the calculation of turn over number (TON). The equation used for the calculation of TON was as follows:

$$TON = \frac{moles\ of\ product\ formed}{(moles\ of\ catalyst\ used)}$$

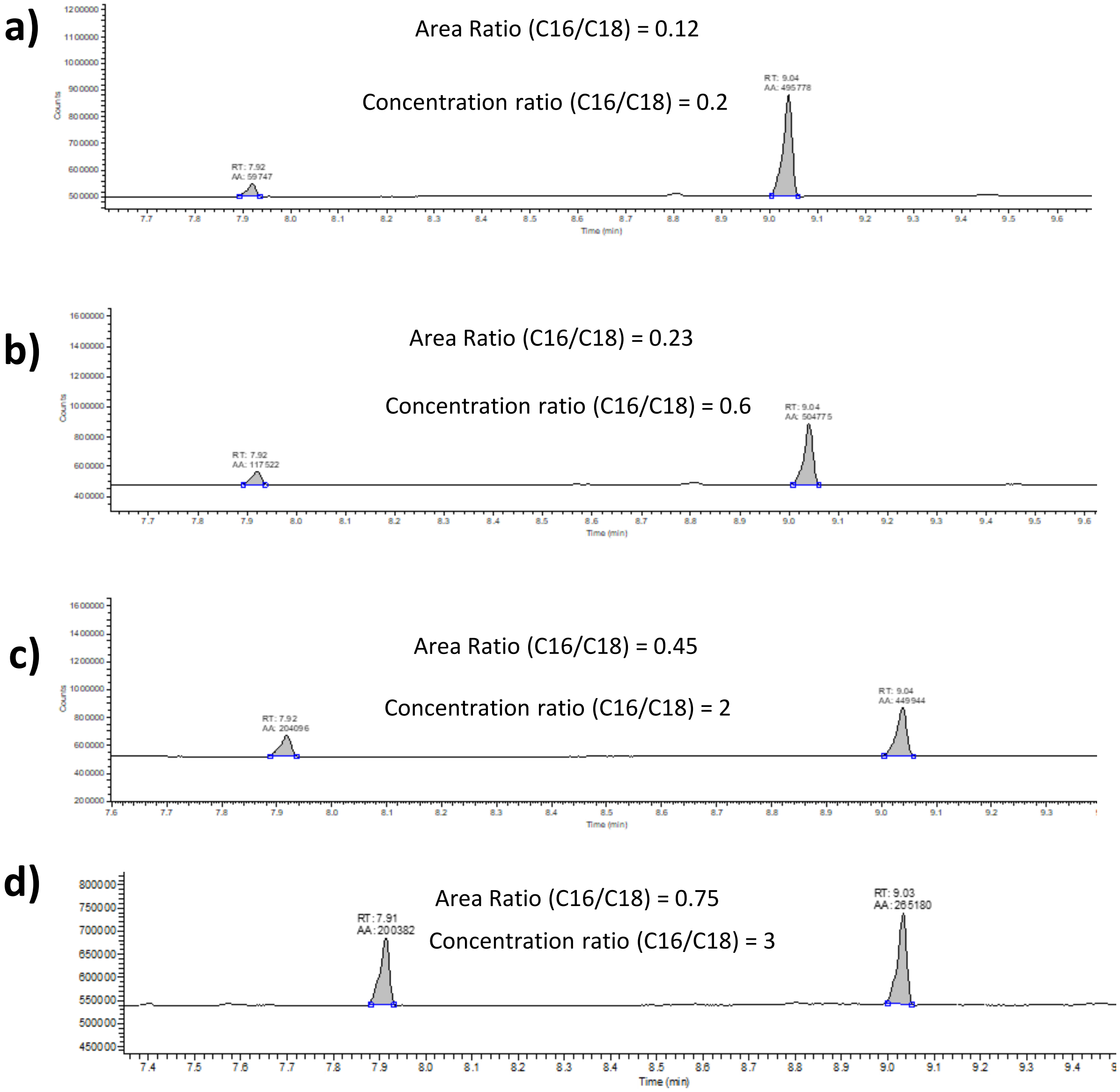

Figure S10: GC chromatographs (a-d) for the substrate (hexadecane) and internal standard (octadecane) and peak area calculation for the calibration curve.

22. Calibration curve for substrate consumption:

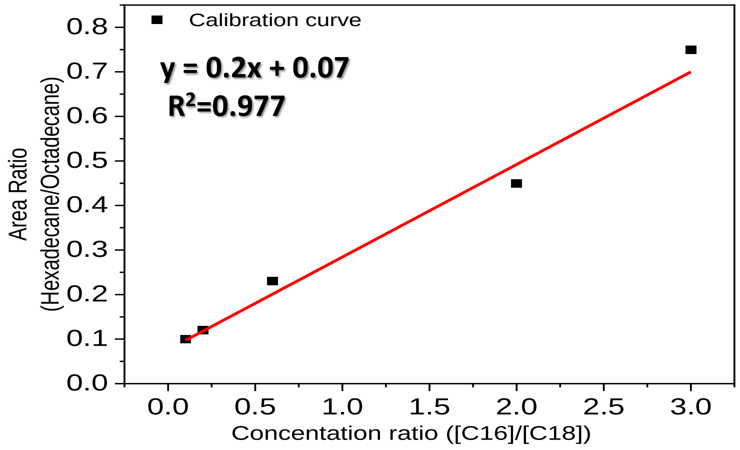

Figure S11: Calibration curve for the hexadecane for the determination of substrate conversion..

23. Product analysis of the catalytic reaction by GC:

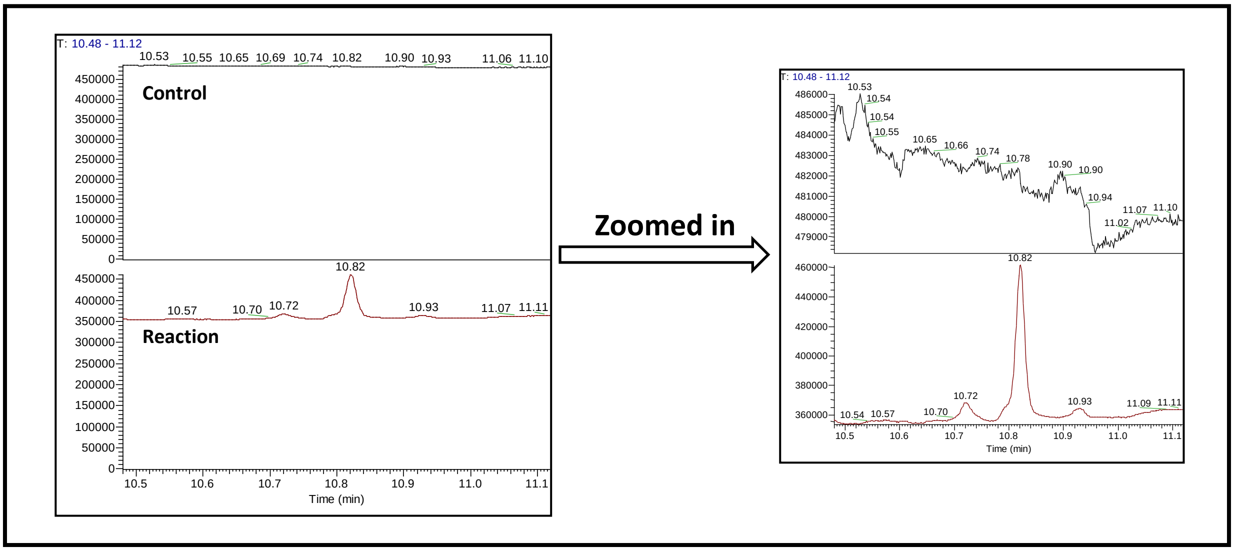

Figure S12: GC-FID chromatograph for the reaction product analysis. Upper trace shows the analysis of control reaction and the lower trace shows the analysis of catalytic reaction catalysed by final mutant LFFTFA of CYP175A1.

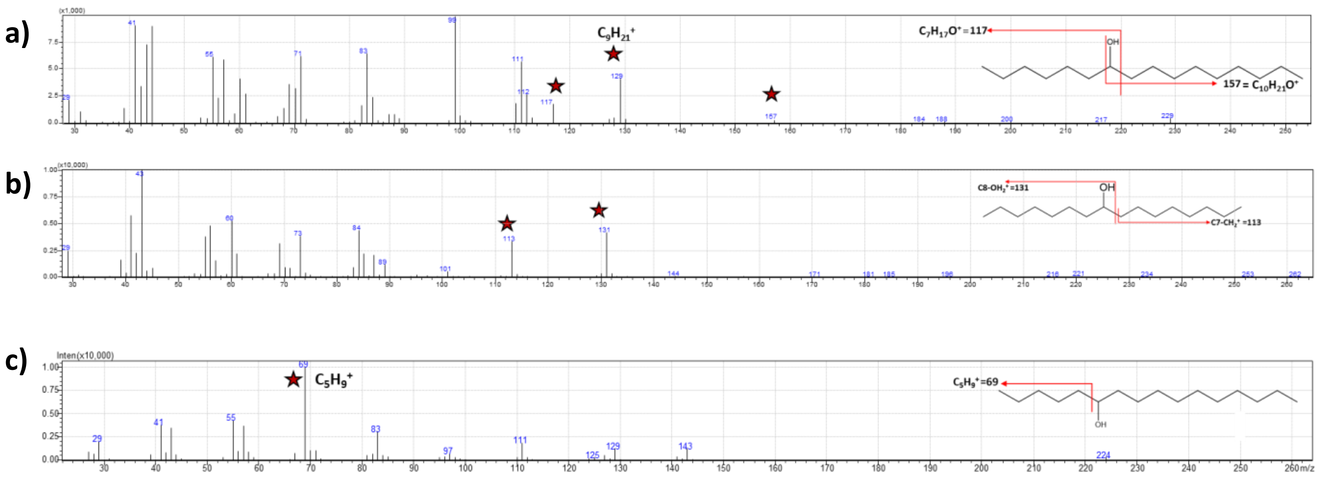

Figure S13: Mass spectrum of the reaction products identified using GC-MS. a) Mass spectrum of 7-Hexadecanol b) Mass spectrum of 8-Hexadecanol c) Mass spectrum of 6-Hexadecanol.

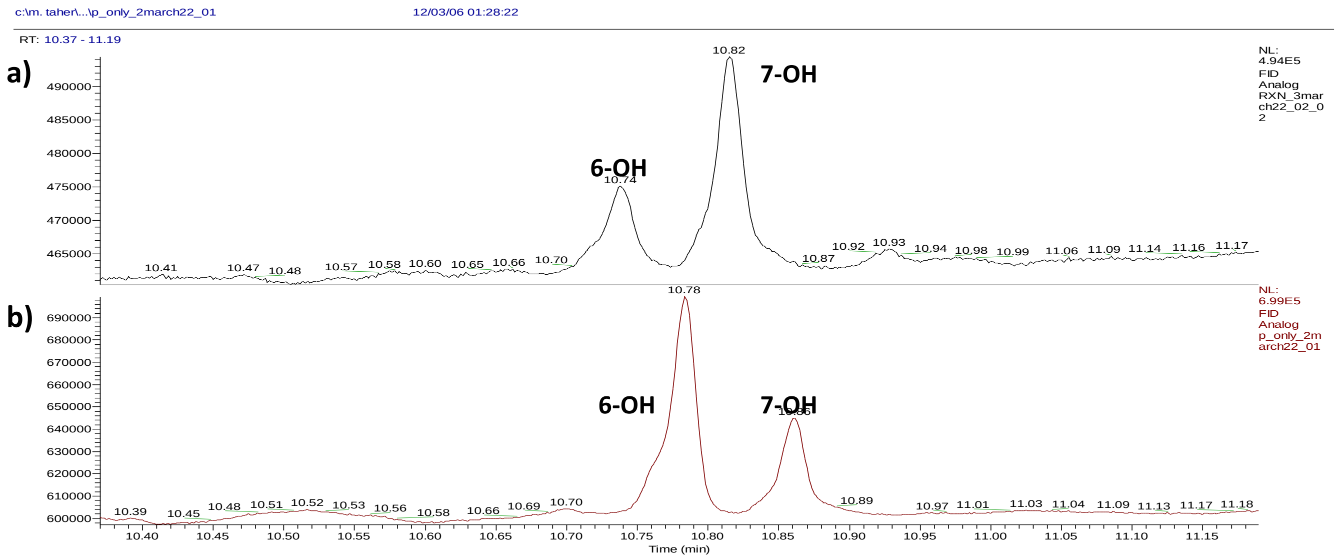

Figure S14: GC-FID chromatogram for the reaction product analysis. a) Chromatogram shows the analysis of reaction catalysed by FA mutant of CYP175A1 b) Chromatogram shows the analysis of catalytic reaction catalysed by FTFA mutant of CYP175A1.

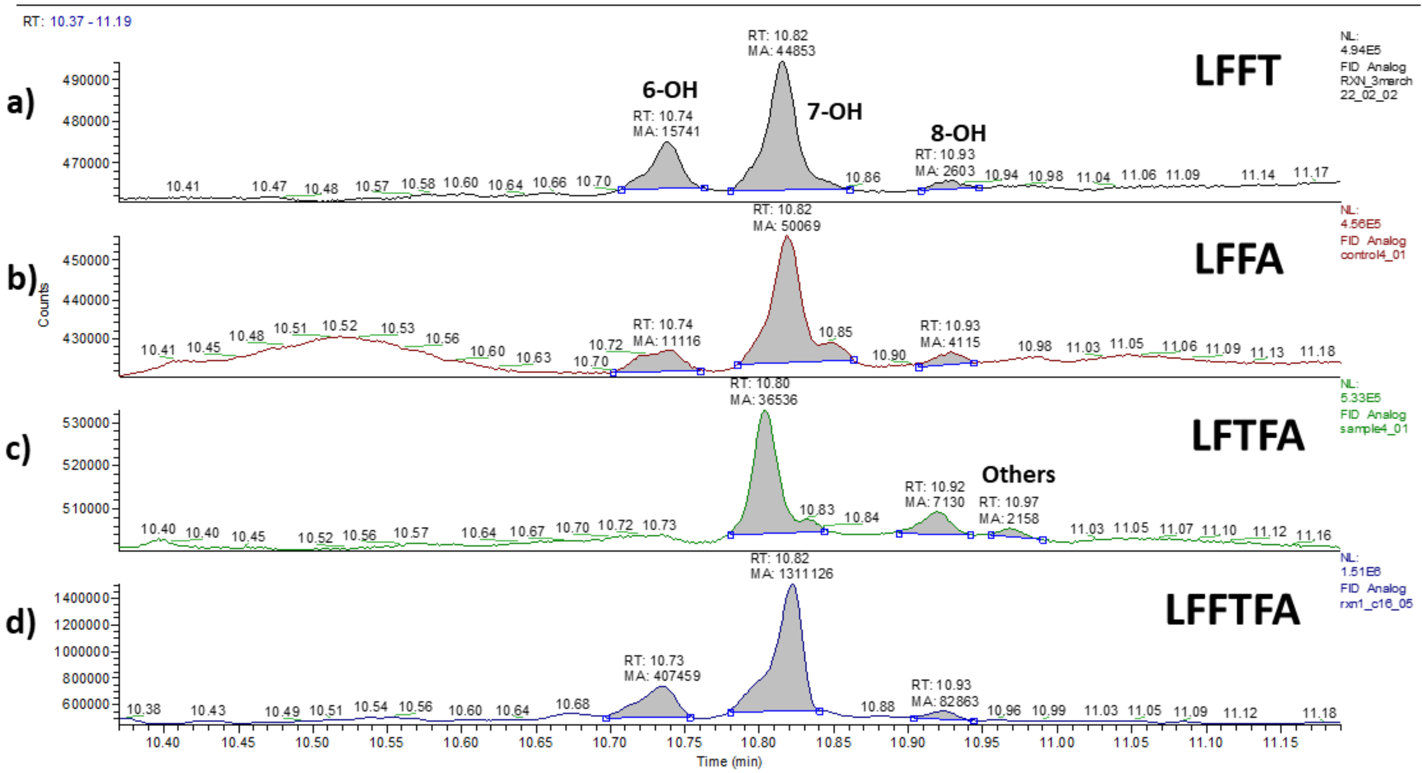

Figure S15: GC-FID chromatogram for the reaction product analysis. Chromatogram shows the product distribution of the reaction catalysed by a) QYLV, b) QYWI, c) QYVWI and d) QYLVWI mutants of CYP175A1.

24. CD measurements:

Circular Dichroism spectroscopy experiments were carried out using a JASCO J-810 spectropolarimeter with a Peltier cooled sample holder. Far-UV circular dichroism spectra for wild type and the mutants of CYP175A1 were taken using a 10 μM protein solution in 50mM Tris buffer (pH 8.0) with an airtight capped 1 mm path length cell. The far-UV CD spectrum arises due to polarization effects in the amide bond transitions. The circular dichroism (CD) spectra of all the variants of CYP175A1 in the far-UV region (Figure S16) show typical double hump nature, characteristic of predominantly helical globular protein. The far-UV CD spectra of the proteins at 25 °C shown in Figure S16 are almost identical in all the six forms of the protein with large negative ellipticity having peaks at 220 nm and 208 nm, suggesting that the secondary structure of the protein was unchanged on performing the mutation. All the experiments were performed three times. The typical secondary structural motif have been found to be associated with the characteristic pattern of CD spectra in the far-UV region. Monitoring the CD spectra in this region is a well-established method to monitor the change in the secondary structure of the enzyme and consequently different algorithms[18] have been developed to analyse the secondary structure from the far UV-CD spectra of the proteins. CD temperature dependent data were analysed considering the two-state transition model between folded (N) and unfolded state (U) of the protein.

$$N \rightleftharpoons \mathrm{U} \quad \text{.........(1)}$$

The free energy change of the unfolding (ΔG_U) can be defined as:

$$\Delta G_U = -R.T \ln K_U = -R.T \ln(\frac{f_U}{1-fU}) \quad \text{.......... (2)}$$

Here R is the universal gas constant, K_U is the equilibrium constant for the unfolding, and f_U is the fraction of unfolded protein obtained from the following expression:

$$f_U = \frac{y_0 - yN + mN.x}{(y_U + mU.x) - (yN + mN.x)} \quad \text{.......(3)}$$

Where y_0 is the observed CD value in mili-degree (mDeg) at a given instance. y_N and y_U represents the intercepts, and m_N and m_U are the slopes of the native and unfolded baselines respectively.

The following Equation (4) was used to fit the raw CD data obtained for temperature-dependent unfolding of the wild type and mutant CYP175A1 proteins by using the data analysis program, origin from origin lab.

$$y = \frac{(y_n + m_n.T) + (y_u + m_u.T).e^{(\frac{(-\Delta Hm(1 - T/Tm) + \Delta Cp(T - Tm - T.\ln(T/Tm)))}{(R.T)})}}{1 + e^{(\frac{(-\Delta Hm(1 - T/Tm) + \Delta Cp(T - Tm - T.\ln(T/Tm)))}{(R.T)})}} \quad \text{......(4)}$$

Tm is the mid point transition temperature for the protein unfolding. The change in enthalpy of unfolding and the change in heat capacity at constant pressure are defined as ΔH_m,ΔC_p respectively. R is universal gas constant, 8.314 $\mathrm{Jmol^{-1}K^{-1}}$in S.I unit.

The stability curve for wild type and mutant CYP175A1 were obtained for the secondary unfolding by nonlinear square analysis of the above data ($G_T{}^{Aq}$) as a function of temperature by fitting to the Gibbs-Helmholtz equation (Equation 5).

$$\Delta G_T^{Aq} = \Delta H_m \left(1 - \frac{T}{T_m}\right) + \Delta C_p \left(T - T_m - T \ln\left(\frac{T}{T_m}\right)\right) \quad \text{....... (5)}$$

The different thermodynamic parameters such as ΔHm,, could be obtained for secondary and heme tertiary structure unfolding from the above analysis of their respective ellipticity vs T curve. The temperature of maximum stability (T_S) was obtained from the stability curve here slope of the tangent is zero i.e. $\frac{\partial \Delta G(T)}{\partial T} = 0$.

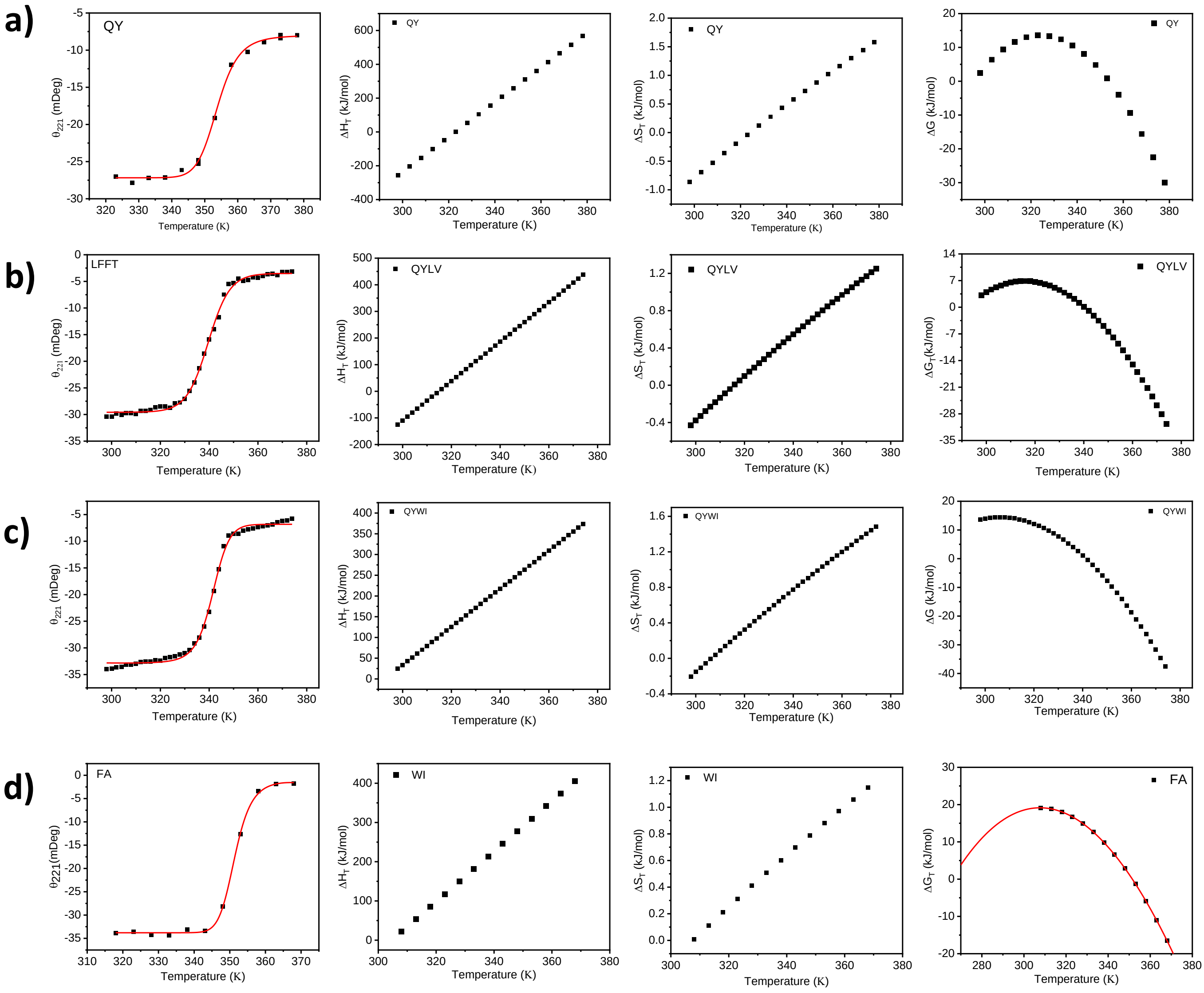

Figure S16: Thermal induced unfolding profiles for secondary structure of the mutants of CYP175A1 obtained from CD at 221 nm in 50 mM Tris buffer, pH 8. ΔH_T, ΔS_T and ΔG_T curves plotted for secondary structure of mutants of CYP175A1. Each point in the thermodynamic parameter curves represents the value at corresponding temperature. The solid line represents non-linear curve fits to Gibbs–Helmholtz equation.

S. No.	Enzyme	T_m (°C)	ΔH_m (kj/mol)	ΔC_p (kJ mol^{-1} K^{-1})
1	WT_CYP175A1	88±2	297±31	4.8±0.5
2	LF_CYP175A1	80.9±2	319.3±20	10.2±0.5
3	FA_CYP175A1	78.5±1	300±24	6.7±0.6
5	LFFA_CYP175A1	68.4±1	275.11±16	7.42±0.6
6	LFFT_CYP175A1	67.3±1	188.5±34	7.4±0.5
7	FTFA_CYP175A1	66.5±2	326.35±19	8.5±0.4
8	LYTFA_CYP175A1	63±2	219.6±27	10.4±0.9
9	LFFTFA_CYP175A1	66±1	299.6±21	12.5±1

Table S7: Thermodynamic parameters for secondary structures of wild type and mutants of CYP175A1, from Thermus thermophilus. T_m, H_m and C_P were determined from non-linear curve fitting of the respective protein stability curves to the Gibbs–Helmholtz equation.

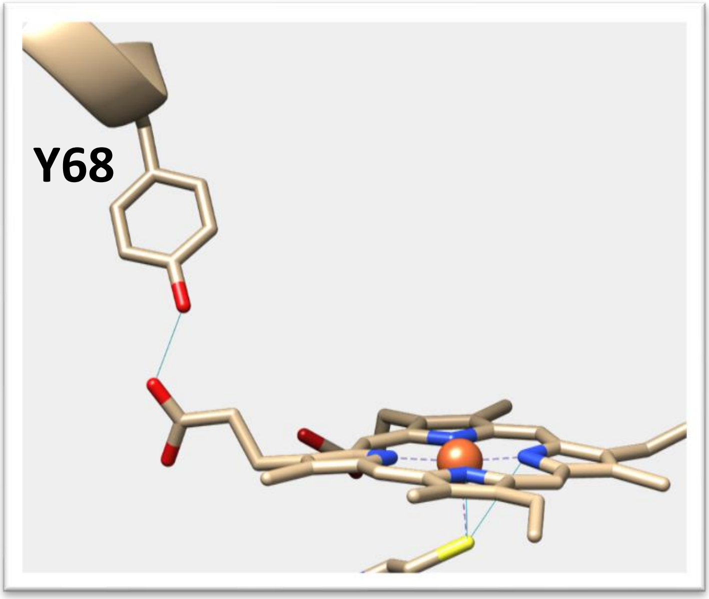

Figure S17: Hydrogen bonding between Y68 (O-H) and heme group (C=O).

25. Supporting video 1 (VS1):

VS1 shows expulsion of the substrate out of the active pocket of the wild-type enzyme.

26. Supporting video 2 (VS2):

VS2 shows low water content around the active pocket of the mutant enzyme (LF) substrate complex.

27. Supporting video 3 (VS3):

VS3 shows high water content around the active pocket of the mutant enzyme (LFFT) substrate (C16) complex.

28. References:

1) G Verdonk, M. L., Cole, J. C., Hartshorn, M. J., Murray, C. W., & Taylor, R. D. (2003). Improved protein–ligand docking using GOLD. Proteins: Structure, Function, and Bioinformatics, 52(4), 609-623.

2) Eldridge, M. D.; Murray, C. W.; Auton, T. R.; Paolini, G. V.; Mee, R. P. Empirical scoring functions: I. The development of a fast empirical scoring function to estimate the binding affinity of ligands in receptor complexes. J. Comput.-Aided Mol. Des. 1997, 11, 425−445

3) Korb, O., Stützle, T., & Exner, T. E. (2009). Empirical scoring functions for advanced protein-ligand docking with PLANTS. Journal of chemical information and modeling, 49(1), 84–96.

4) Tian, C.; Kasavajhala, K.; Belfon, K. A. A.; Raguette, L.; Huang, H.; Migues, A. N.; Bickel, J.; Wang, Y.; Pincay, J.; Wu, Q. Ff19SB: Amino-Acid-Specific Protein Backbone Parameters Trained against Quantum Mechanics Energy Surfaces in Solution. *J. Chem. Theory Comput.* **2019**, *16*, 528–552.

5) Shahrokh K, Orendt A, Yost GS, Cheatham TE 3rd. Quantum mechanically derived AMBER-compatible heme parameters for various states of the cytochrome P450 catalytic cycle. J Comput Chem. 2012 ;33:119-33.

6) Bayly, C. I.; Cieplak, P.; Cornell, W.; Kollman, P. A. A Well-Behaved Electrostatic Potential Based Method Using Charge Restraints for Deriving Atomic Charges: The RESP Model. J. Phys. Chem. 1993, 97, 10269–10280.

7) Izaguirre, J. A.; Catarello, D. P.; Wozniak, J. M.; Skeel, R. D. Langevin Stabilization of Molecular Dynamics. J. Chem. Phys. 2001, 114, 2090–2098.

8) Berendsen, H. J. C.; Postma, J. P. M. van; Van Gunsteren, W. F.; DiNola, A.; Haak, J. R. Molecular Dynamics with Coupling to an External Bath. J. Chem. Phys. 1984, 81, 3684–3690.

9) Darden, T.; York, D.; Pedersen, L. Particle Mesh Ewald: An N· Log (N) Method for Ewald Sums in Large Systems. J. Chem. Phys. 1993, 98, 10089–10092.

10) Salomon-Ferrer, R.; Gotz, A. W.; Poole, D.; Le Grand, S.; Walker, R. C. Routine Microsecond Molecular Dynamics Simulations with AMBER on GPUs. 2. Explicit Solvent Particle Mesh Ewald. J. Chem. Theory Comput. 2013, 9, 3878–3888.

11) Metz, S.; Kästner, J.; Sokol, A. A.; Keal, T. W.; Sherwood, P. ChemShell-a Modular Software Package for QM/MM Simulations. Wiley Interdiscip. Rev. Comput. Mol. Sci. 2014, 4, 101–110.

12) Balasubramani, S. G.; Chen, G. P.; Coriani, S.; Diedenhofen, M.; Frank, M. S.; Franzke, Y. J.; Furche, F.; Grotjahn, R.; Harding, M. E.; Hättig, C.; Hellweg, A.; Helmich-Paris, B.; Holzer, C.; Huniar, U.; Kaupp, M.; Marefat Khah, A.; Karbalaei Khani, S.; Müller, T.; Mack, F.; Nguyen, B. D.; Parker, S. M.; Perlt, E.; Rappoport, D.; Reiter, K.; Roy, S.; Rückert, M.; Schmitz, G.; Sierka, M.; Tapavicza, E.; Tew, D. P.; Van Wüllen, C.; Voora, V. K.; Weigend, F.; Wodyński, A.; Yu, J. M. TURBOMOLE: Modular Program Suite for Ab Initio Quantum-Chemical and Condensed-Matter Simulations. J. Chem. Phys. 2020, 152.

13) Ahlrichs, R.; Bär, M.; Häser, M.; Horn, H.; Kölmel, C. Electronic Structure Calculations on Workstation Computers: The Program System Turbomole. Chem. Phys. Lett. 1989, 162, 165–169.

14) Smith, W.; Forester, T. R. DL_POLY_2.0: A General-Purpose Parallel Molecular Dynamics Simulation Package. J. Mol. Graph. 1996, 14, 136–141.

15) Grimme, S. Semiempirical GGA-type density functional constructed with a long-range dispersion correction. J. Comput. Chem. 2006, 27, 1787−1799.

16) Ramanan, R., Dubey, K. D., Wang, B., Mandal, D., & Shaik, S. (2016). Emergence of function in P450-proteins: a combined quantum mechanical/molecular mechanical and molecular dynamics study of the reactive species in the H2O2-dependent cytochrome P450SPα and Its regio-and enantioselective hydroxylation of fatty acids. Journal of the American Chemical Society, 138(21), 6786-6797.

17) Omura, T., & Sato, R. (1964). The carbon monoxide-binding pigment of liver microsomes. I. Evidence for its hemoprotein nature. J biol Chem, 239(7), 2370-2378.

18) Greenfield, N. J. (2006). Using circular dichroism spectra to estimate protein secondary structure. Nature protocols, 1(6), 2876-2890.
